# Supplementary material for: Spinning sugars in antigen biosynthesis: characterization of the Coxiella burnetii and Streptomyces griseus TDP-sugar epimerases
Source: J Biol Chem. 2022 Apr 6;298(5):101903. doi: 10.1016/j.jbc.2022.101903 (PMC9095892; doi:10.1016/j.jbc.2022.101903)
Supplement: Supplemental Tables S1–S5 and Figures S1–S6 [file mmc1.docx]

**Supplementary information**

**Table S1: Sequence identities and root mean squared deviation values for close homologues of CBU1838 and StrM.** Sequence identities are shown below the diagonal as percentages; RMSD values for alpha carbons are shown above the diagonal in Å. Well-characterised RmlC orthologues with structures were aligned using Geneious Prime v. 2022.1.1 (https://www.geneious.com). The MUSCLE algorithm was used with default parameters (10). The TDP-6-deoxy-d-*xylo*-4-hexulose 3’’, 5’’, or double epimerases were included as StrM and CBU1838 group with these (Figure S5). Sequence identities were calculated using Geneious Prime. Protein structures were cleaned to remove tag sequences; they were superimposed and RMSD calculated using the MatchMaker tool in UCSF ChimeraX v. 1.2 (11). Values are coloured by activity, consistently with Figure S5: TDP-6-deoxy-d-*xylo*-4-hexulose 3’’,5’’-epimerase, green; TDP-6-deoxy-d-*xylo*-4-hexulose 5’’-epimerase, blue; TDP-6-deoxy-d-*xylo*-4-hexulose 3’’-epimerase, vermillion; CBU1838 and StrM (this study), black. Proteins are ordered using the phylogeny in Figure S5. Species for each entry: 3RYK: *Bacillus anthracis* (12); 1EP0: *Methanobacterium thermoautotrophicum* (13); 1DZR: *Salmonella typhimurium* (14); 2IXH: *Pseudomonas aeruginosa* (7); 7PVI: *Coxiella burnetii* (this study); 1NYW: *Streptococcus suis* (5); 2IXC: *Mycobacterium tuberculosis* (7); 1OI6: *Amycolatopsis orientalis* (6); 2C0Z: *Streptomyces niveus* (15); 4HN0: *Streptomyces bikiniensis* (16); 7PWI: *Streptomyces griseus* (this study).

|  | 3RYK | 1EP0 | 1DZR | 2IXH | 7PVI | 1NYW | 2IXC | 1OI6 | 2C0Z | 4HN0 | 7PWI |
| --- | --- | --- | --- | --- | --- | --- | --- | --- | --- | --- | --- |
| 3RYK |  | 1.37 | 1.57 | 1.21 | 1.70 | 2.67 | 1.30 | 1.94 | 1.69 | 1.93 | 2.10 |
| 1EP0 | 42 |  | 2.14 | 1.24 | 2.12 | 4.27 | 3.20 | 2.87 | 2.06 | 2.70 | 2.63 |
| 1DZR | 49 | 48 |  | 1.67 | 1.71 | 2.79 | 1.84 | 2.24 | 1.75 | 2.24 | 2.28 |
| 2IXH | 46 | 47 | 61 |  | 1.15 | 2.43 | 1.41 | 1.78 | 1.60 | 2.35 | 2.30 |
| 7PVI | 37 | 46 | 51 | 53 |  | 3.02 | 2.45 | 2.75 | 2.55 | 3.04 | 2.42 |
| 1NYW | 25 | 25 | 28 | 31 | 27 |  | 3.34 | 2.97 | 3.14 | 3.34 | 3.33 |
| 2IXC | 32 | 36 | 36 | 37 | 35 | 27 |  | 1.59 | 0.907 | 1.59 | 1.23 |
| 1OI6 | 27 | 30 | 32 | 35 | 30 | 24 | 40 |  | 1.30 | 1.85 | 2.06 |
| 2C0Z | 32 | 39 | 36 | 39 | 31 | 24 | 47 | 44 |  | 1.07 | 1.22 |
| 4HN0 | 28 | 31 | 31 | 34 | 31 | 28 | 45 | 43 | 48 |  | 1.19 |
| 7PWI | 36 | 35 | 33 | 32 | 29 | 26 | 48 | 42 | 48 | 53 |  |

**Table S2: Crystallographic data table**

| Protein complex | CBU1838  xylose | CBU1838  TDP | StrM apo | StrM TDP |
| --- | --- | --- | --- | --- |
| Data collection statistics | | | | |
| Beamline | I04 Diamond | I04 Diamond | I03 Diamond | I04-1 Diamond |
| Wavelength (Å) | 0.9795 | 0.9795 | 0.9763 | 0.9159 |
| Space group | C2 | C222_1_ | C222_1_ | C222_1_ |
| Unit Cell Parameters  a, b, c (Å) | 77.0, 84.6,  77.7 | 78.9, 82.2,  163.8 | 42.3, 132.4,  77.6 | 42.4, 131.5,  78.5 |
| α, β, γ (**°)** | 90.0, 113.6,  90.0 | 90.0, 90.0, 90.0 | 90.0, 90.0, 90.0 | 90.0, 90.0, 90.0 |
| Resolution range (Å)^a^ | 51.39 – 1.44  (1.61-1.45) | 81.92-1.87  (2.0-1.87) | 66.18 – 1.34  (1.43 - 1.34) | 50.42-1.90  (1.94-1.90) |
| Total reflections ^a^ | 182,903  (7,987) | 247,730  (12,776) | 230,889  (11,075) | 76,059  (4,895) |
| Unique reflections ^a^ | 54,839 (2,742) | 37,452 (1,874) | 36,479 (1,824) | 17,624 (1,122) |
| Completeness spherical (%)^a^ | 65.9 (11.6)^b^ | 84.8 (25.0)^b^ | 74.0 (20.8)^b^ | 99.4 (99.6) |
| Multiplicity ^a^ | 3.3 (2.9) | 6.6 (6.8) | 6.3 (6.1) | 4.3 (4.4) |
| R*_meas_* (%) ^a,d^ | 5.7 (85.2) | 8.5 (140.0) | 14.2 (249.5) | 24.5 (240.5) |
| <*I>/<σ(I)>* ^a^ | 12.5 (1.5) | 12.5 (1.3) | 5.6 (0.3) | 5.5 (0.7) |
| CC_1/2_ ^a,c^ | 0.999 (0.585) | 0.998 (0.67) | 0.996 (0.304) | 0.991 (0.312) |
| Wilson B-factor ^e^ (Å^2^) | 27.9 | 50.7 | 17.9 | 30.7 |
| Refinement statistics | | | | |
| *R_work_* | 0.168 | 0.209 | 0.201 | 0.203 |
| *R_free_* | 0.199 | 0.237 | 0.234 | 0.249 |
| No. of protomers in a.u. | 2 | 2 | 1 | 1 |
| Number of atoms |  |  |  |  |
| Protein | 3,540 | 3,324 | 1,727 | 1648 |
| Ligands | 53 | 100 | - | 25 |
| Solvent | 459 | 292 | 228 | 180 |
| Number of protein residues | 390 | 380 | 200 | 203 |
| RMS bond lengths (Å) | 0.008 | 0.011 | 0.009 | 0.006 |
| RMS bond angles (°) | 1.45 | 1.59 | 1.81 | 1.40 |
| Ramachandran  favoured (%)^f^ | 99.48 | 97.61 | 97.98 | 98.51 |
| Ramachandran outliers (%)^f^ | 0.0 | 0.27 | 0.0 | 0.0 |
| Clashscore ^e^ | 9.5 | 7.9 | 6.6 | 5.94 |
| Average B-factor protein (Å^2^) | 22.3 | 41.2 | 13.9 | 26.8 |
| Average B-factor ligands (Å^2^) | 30.1 | 75.5 | - | 52.4 |
| Average B-factor solvent (Å^2^) | 35.2 | 48.5 | 26.6 | 36.7 |
| RCBS PDB code | 7PVI | 7PWB | 7PWI | 7PWH |

^a^Values for the highest resolution shell are given in parentheses.

^b^ Values are given for data subjected to anisotropic ellipsoidal truncation using the STARANISO server (1)

^c^ *R_meas_* **=** Σ_h_[m/(m - 1)]^1/2^ Σ|_i_*I_h,i_* - <*I_h_*>|/ Σ*_h_* Σ_i_*I_h,i._*

^d^ CC_1/2_ is defined in (2).

^e^ Wilson B-factor was estimated by SFCHECK (3).

^f^ The Ramachandran statistics and clashscore statistics were calculated using MOLPROBITY (4).

**Table S3: Kinetic data for RmlC paralogues from diverse species.**

| Species | *k_cat_* (s^-1^) | *K_M_* for TDP-6-deoxy-d-*xylo*-4-hexulose (μM) | Temperature (° C) | References |
| --- | --- | --- | --- | --- |
| *S. suis* | 10.4 ± 0.3 | 29 ± 3 | 21 °C | (5,6) |
| *S. enterica* serovar Typhimurium | 19.2 ± 0.5 | 81 ± 8 | 21 °C | (5,6) |
|  | 39.0 ± 6.6 | 710 ± 170 | 25 °C | (7,8) |
| *M. tuberculosis* | 18.0 ± 0.9 | 211 ± 43 | 25 °C | (9) |
| *A. thermoaerophilus* | 2.0 ± 0.7 | 62 ± 6 | 25 °C | (8) |
| *E. coli* | 5.5 ± 0.1 | 830 ± 40 | 37 °C | This study |
| *S. griseus* StrM | 5.4 ± 0.1 | 600 ± 40 | 37 °C | This study |
| *C. burnetii* CBU1838 | 1.75 ± 0.05 | 2,000 ± 200 | 37 °C | This study |
| *A. orientalis* EvaD | 0.39 ± 0.02 | 632 ± 98 | 37 °C | This study |

**Table S4: Primer sequences.** Primers used in this study were purchased from IDT.

| Primer name | Sequence (5’-3’) |
| --- | --- |
| RmlB_F | TACTTCCAATCCATGAAAATACTTGTTACTGGTGGCGCAGG |
| RmlB_R | TATCCACCTTTACTGTTACTGGCGGCCCTCATAGTTCTGTTCAATCC |
| RmlC_F | TACTTCCAATCCATGAATGTGATTAGAACTGAAATTGAAGATGTGC |
| RmlC_R | TATCCACCTTTACTGTCATGCAATTAATTTTAATCTGATAAGC |
| RmlD_F | TACTTCCAATCCATGAATATCCTCCTTTTTGGCAAAACAGG |
| RmlD_R | TATCCACCTTTACTGTTAAATTGCTGTAGTCGTAAATAATTCATTGAGC |
| RmlD_insC_F | GCAAAGCAGGCATTCCCCTTGCACTCAACAAGCTC |
| RmlD_insC_R | GAGCTTGTTGAGTGCAAGGGGAATGCCTGCTTTGC |
| CBU1838_F | TACTTCCAATCCATGCCGTTTGAATTTCAAAAAATGCTC |
| CBU1838_R | TATCCACCTTTACTGTTAAGAGCCATGATACTGCGC |

**Table S5: Crystallisation and soaking conditions.**

|  | Structures | | | |
| --- | --- | --- | --- | --- |
| Complex | CBU1838  xylose | CBU1838  TDP | StrM apo | StrM TDP |
| RCSB PDB code | 7PVI | 7PWB | 7PWI | 7PWH |
| Protein stock concentration (mg/mL) | 4.5 (co-crystallised with 30 % (w/v) xylose) | 4.5 (co-crystallised with 10 mM TDP) | 5.85 | 5.85 |
| Precipitant mixture | 25 % (w/v) PEG 3350, 100 mM Bis-Tris pH 5.5 | 8 % (w/v) PEG 4000, 0.1 M sodium acetate pH 4.6 | 20 % (w/v) PEG 6000, 100 mM CaCl_2_, 50 mM HEPES pH 7.0 | 20 % (w/v) PEG 6000, 100 mM CaCl_2_, 50 mM HEPES pH 7.0 |
| Ratio of protein: precipitant: seeds | 3:2:1 (1:1000 seed stock) | 3:2:1 (1:100 seed stock) | 1:1 | 1:1 |
| Cryoprotectant solution | 100 mM  (NH_4_)_2_SO_4_, 8 % (w/w)  PEG 8000, 30 %  (w/v) xylose | 7 % (w/v) PEG 4000, 30 % (v/v) PEG 400, 5 mM TDP, 29 mM citrate pH 4.5 | 10 % (w/v) PEG  6,000, 30 % (v/v)  PEG 300, 100  mM CaCl_2_, 50  mM Tris pH 7.0 | 10 % (w/v) PEG  6,000, 30 % (v/v)  PEG 300, 100  mM CaCl_2_, 50  mM Tris pH 7.0, 10 mM TDP |
| Soaking time (min) | 1 | 1 | 1 | 6 |


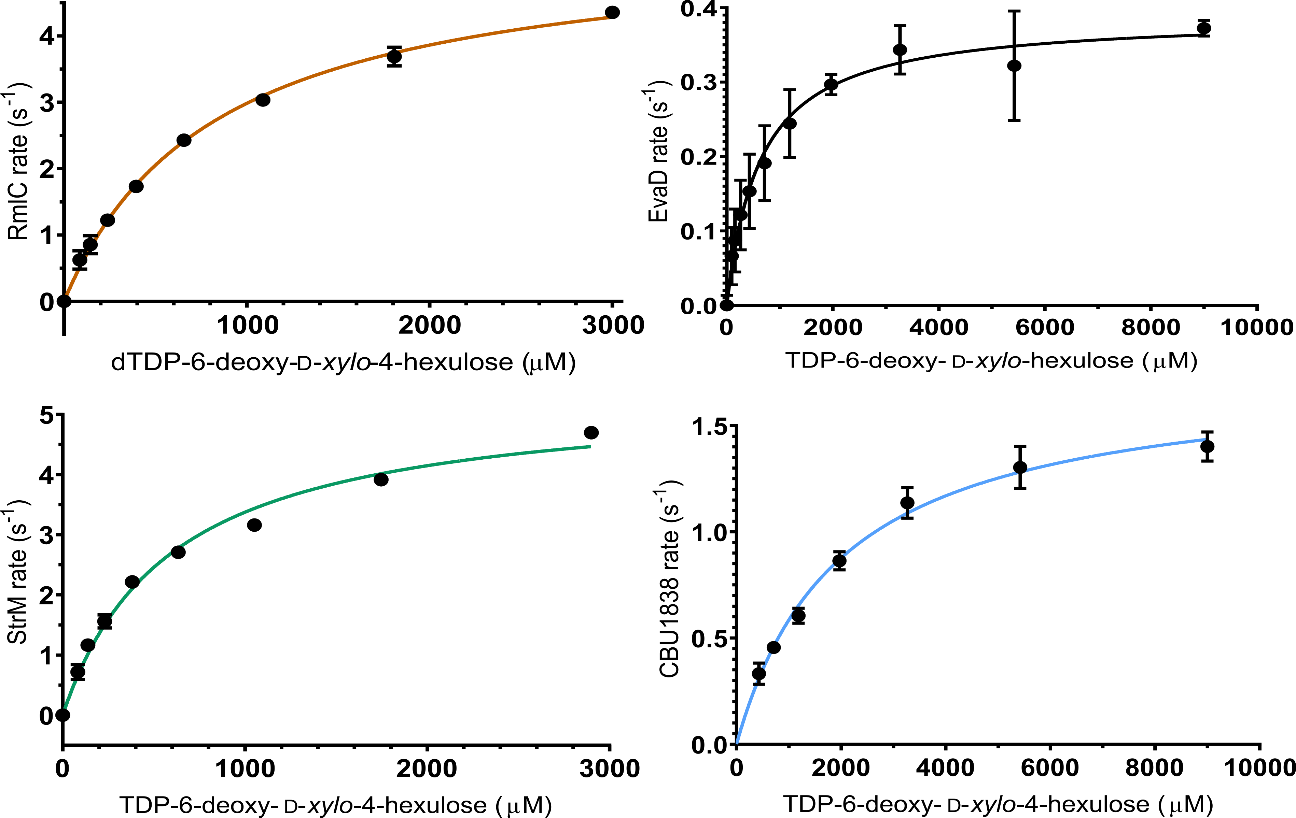


**Figure S1. Kinetic activity of TDP-sugar epimerases.**

RmlC and StrM were used at 0.2 µM, CBU1838 was used at 0.6 µM, and *Ao*EvaD at 1.0 µM, each with 0.8 µM RmlD, 350 µM NADPH, 20 mM MgCl_2_, and 50 mM HEPES pH 7.0. Due to a slight lag phase, the steepest slope from each substrate concentration replicate was chosen for rate analysis. For RmlC and StrM at the highest substrate concentration, completion was not reached after 300-500 s. For CBU1838 completion was reached after 50-550 s, for *Ao*EvaD this was doubled. Data were fitted to the Michaelis-Menten non-linear regression analysis equation. Data were analysed using GraphPad Prism v8.1.2. Three experimental replicates were taken for each data point, and error bars represent the standard error in the mean.

**Figure S2: ChmJ is not capable of 3’’,5’’ double epimerisation of TDP-6-deoxy-d-*xylo*-4-hexulose.** RmlC and StrM were used at 0.4 µM, whilst ChmJ was used at 1.6 µM, each with 0.4 µM each RmlB and RmlD, 5 μM NAD^+^, 350 µM NADH, 500 μM MgCl_2_, 150 μM TDP-glucose and 50 mM HEPES pH 7.5. Whilst RmlC and StrM consume all the TDP-sugar substrate, ChmJ does not cause any turnover. The observed rate is at the limit of detection of the assay (10^-5^ AU s^-1^), which is limited by the natural breakdown of NADPH at 37° C. Data were analysed using GraphPad Prism v8.1.2.

**Figure S3: Rate of RmlC paralogues monitored by NMR.** RmlC paralogues were incubated with purified TDP-6-deoxy-d-*xylo*-4-hexulose. 38 nM *Ec*RmlC, 46 nM *Sg*StrM, 177 nM CBU1838, or 530 nM *Ao*EvaD was used, with more enzyme used for the less active enzymes to ensure that a detectable rate was observed. The loss of proton signal at the 3’’ and 5’’ positions in the sugar was monitored by NMR. The rate was monitored every 5-10 minutes over 90 minutes to calculate an initial rate.


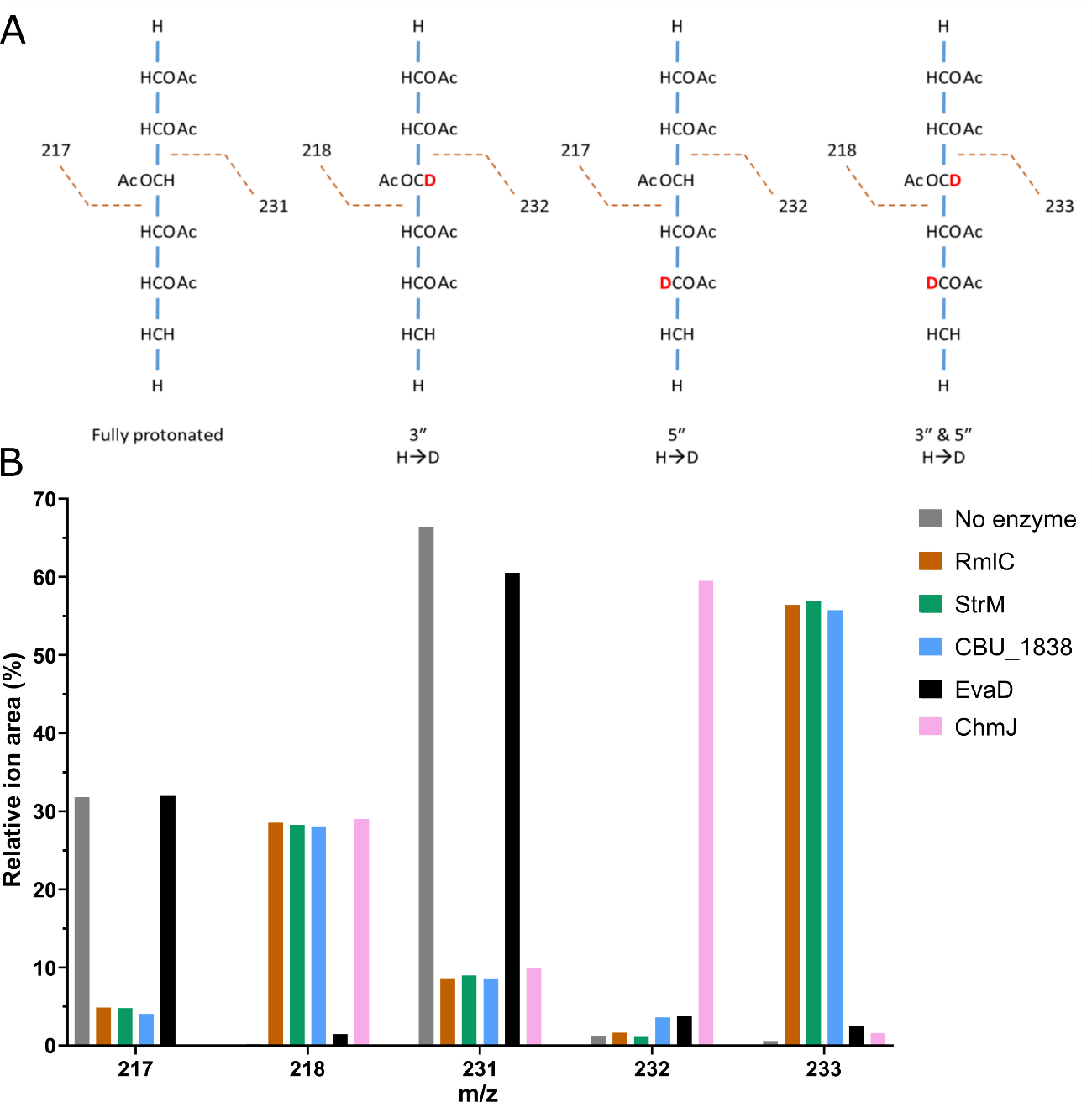


**Figure S4: Deuterium incorporation into TDP-6-deoxy-D-xylo-4-hexulose measured by GC-MS.** 0.5 mg TDP-6-deoxy-d-*xylo*-4-hexulose was incubated in deuterated buffer alone, or with the addition of 1 μM of *Ec*RmlC, *Sg*StrM, CBU1838, *Ao*EvaD or *Sb*ChmJ in 250 μL. After 18 h alditol acetates were prepared following the methods of (17-19). **A.** The characteristic fragmentation products of the original sample and the mono/bi-substituted products. This highlights that the peak at m/z 233 is characteristic of the double substituted product; an ion pair at 218/232 m/z is characteristic of a 3’’ single substitution; and an ion pair at 217/232 m/z is characteristic of a 5’’ single substitution. **B**. Observed products after incubation with enzymes for 18 h. At the test concentrations, RmlC, StrM and CBU1838 show almost complete conversion to the 3’’,5’’ substituted product. EvaD shows a low level of substitution at both 3’’ and 5’’ positions. ChmJ shows almost complete substitution at the 3’’ position, but little substitution at the 5’’ position.


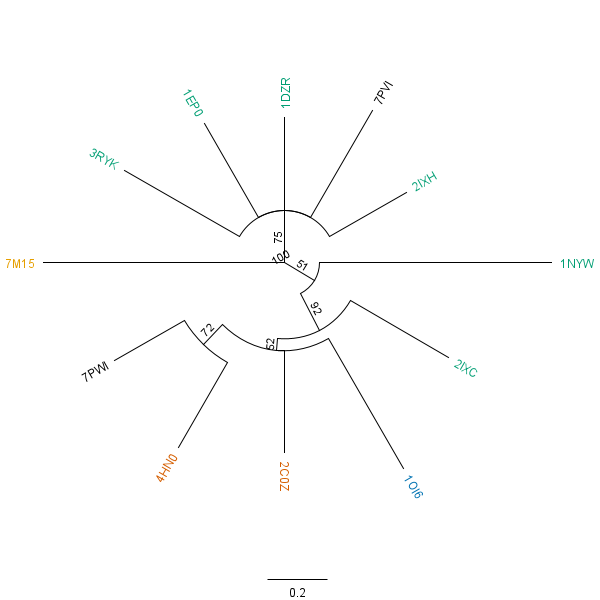


**Figure S5: Phylogenetic tree of RmlC orthologues.** Well-characterised RmlC orthologues with structures were aligned using Geneious Prime v. 2022.1.1 (https://www.geneious.com). The MUSCLE algorithm was used with default parameters (10). The TDP-6-deoxy-d-*xylo*-4-hexulose 3’’, 5’’, or double epimerases were included as StrM and CBU1838 group with these; Cj1430 was included as an outgroup. A phylogenetic tree was prepared using the Geneious tree builder, setting Cj1430 as the outgroup, and re-sampling 100 times. Nodes are coloured by activity: TDP-6-deoxy-d-*xylo*-4-hexulose 3’’,5’’-epimerase, green; TDP-6-deoxy-d-*xylo*-4-hexulose 5’’-epimerase, blue; TDP-6-deoxy-d-*xylo*-4-hexulose 3’’-epimerase, vermillion; GDP-D-glycero-4-keto-D-lyxo-heptose-3’’,5’’-epimerase, orange; CBU1838 and StrM (this study), black. Values at branches indicate the number of times that this grouping was sampled out of 100 (i.e. confidence in this part of the phylogeny).


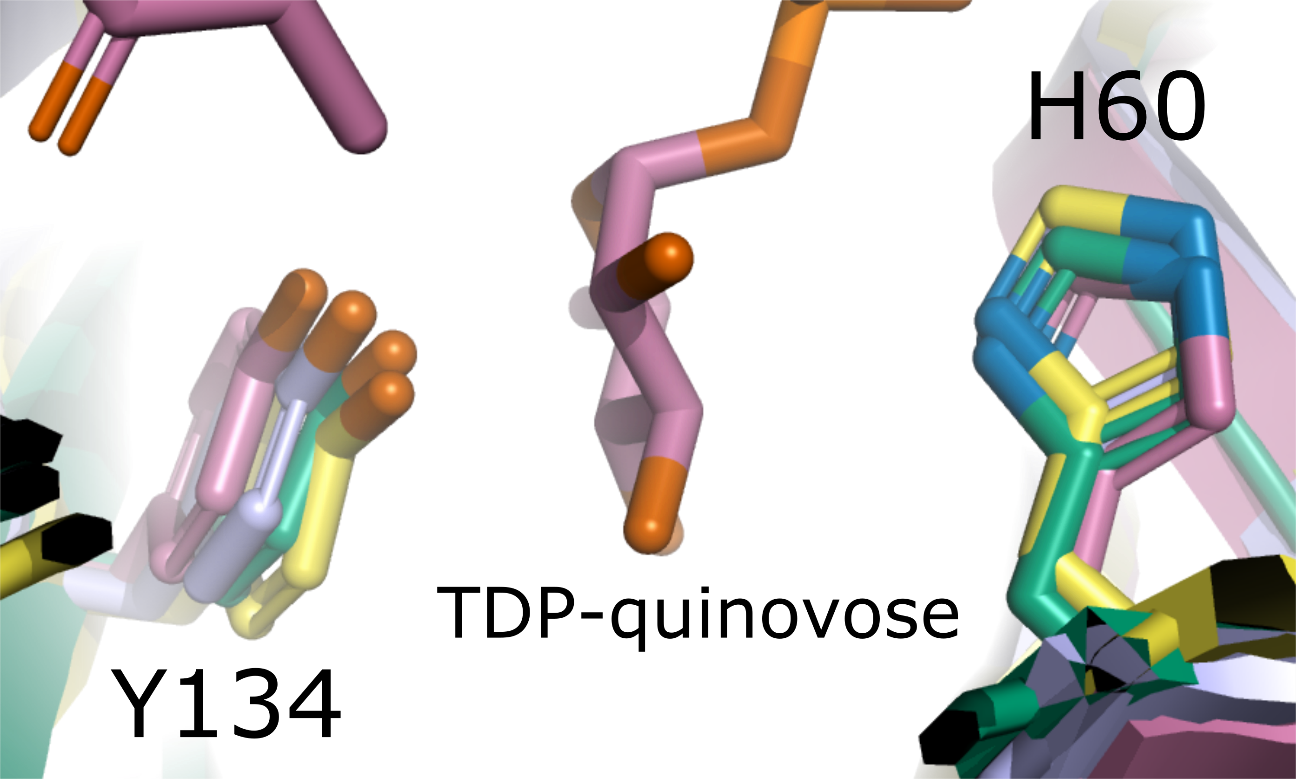


**Figure S6: Key StrM and CBU1838 catalytic residues retain the conformation observed in other paralogues.** The proposed catalytic acid and base Y134 and H60 are shown from the structures of CBU1838 (sea-green) and StrM (yellow) in comparison to the structures of *Mt*RmlC complexed to TDP-rhamnose (2IXC; light blue; (7)) and *Sb*ChmJ complexed to TDP-quinovose (4HMZ; rose; (16)). The TDP-quinovose and catalytic residues are shown as sticks; all other ligands have been removed. Protein backbones are shown as cartoons. Colours: nitrogen, blue; oxygen, red; phosphorus, orange. Structures were superimposed and the image generated using PyMOL v. 2.3.4.

**References**

1. Tickle, I. J., Flensburg, C., Keller, P., Paciorek, W., Sharff, A., Vonrhein, C., and Bricogne, G. (2018) STARANISO. Global Phasing Ltd.

2. Karplus, P. A., and Diederichs, K. (2012) Linking crystallographic model and data quality. *Science* **336**, 1030-1033

3. Vaguine, A. A., Richelle, J., and Wodak, S. J. (1999) SFCHECK: a unified set of procedures for evaluating the quality of macromolecular structure-factor data and their agreement with the atomic model. *Acta Crystallogr D Biol Crystallogr* **55**, 191-205

4. Williams, C. J., Headd, J. J., Moriarty, N. W., Prisant, M. G., Videau, L. L., Deis, L. N., Verma, V., Keedy, D. A., Hintze, B. J., Chen, V. B., Jain, S., Lewis, S. M., Arendall, W. B., 3rd, Snoeyink, J., Adams, P. D., Lovell, S. C., Richardson, J. S., and Richardson, D. C. (2018) MolProbity: More and better reference data for improved all-atom structure validation. *Protein Sci* **27**, 293-315

5. Dong, C., Major, L. L., Allen, A., Blankenfeldt, W., Maskell, D., and Naismith, J. H. (2003) High-resolution structures of RmlC from *Streptococcus suis* in complex with substrate analogs locate the active site of this class of enzyme. *Structure* **11**, 715-723

6. Merkel, A. B., Major, L. L., Errey, J. C., Burkart, M. D., Field, R. A., Walsh, C. T., and Naismith, J. H. (2004) The position of a key tyrosine in dTDP-4-Keto-6-deoxy-D-glucose-5-epimerase (EvaD) alters the substrate profile for this RmlC-like enzyme. *J Biol Chem* **279**, 32684-32691

7. Dong, C., Major, L. L., Srikannathasan, V., Errey, J. C., Giraud, M. F., Lam, J. S., Graninger, M., Messner, P., McNeil, M. R., Field, R. A., Whitfield, C., and Naismith, J. H. (2007) RmlC, a C3' and C5' carbohydrate epimerase, appears to operate via an intermediate with an unusual twist boat conformation. *J Mol Biol* **365**, 146-159

8. Graninger, M., Kneidinger, B., Bruno, K., Scheberl, A., and Messner, P. (2002) Homologs of the Rml enzymes from *Salmonella enterica* are responsible for dTDP-beta-L-rhamnose biosynthesis in the gram-positive thermophile Aneurinibacillus thermoaerophilus DSM 10155. *Appl Environ Microbiol* **68**, 3708-3715

9. Sivendran, S., Jones, V., Sun, D., Wang, Y., Grzegorzewicz, A. E., Scherman, M. S., Napper, A. D., McCammon, J. A., Lee, R. E., Diamond, S. L., and McNeil, M. (2010) Identification of triazinoindol-benzimidazolones as nanomolar inhibitors of the *Mycobacterium tuberculosis* enzyme TDP-6-deoxy-d-xylo-4-hexopyranosid-4-ulose 3,5-epimerase (RmlC). *Bioorg Med Chem* **18**, 896-908

10. Edgar, R. C. (2021) MUSCLE v5 enables improved estimates of phylogenetic tree confidence by ensemble bootstrapping. *bioRxiv*, 2021.2006.2020.449169

11. Goddard, T. D., Huang, C. C., Meng, E. C., Pettersen, E. F., Couch, G. S., Morris, J. H., and Ferrin, T. E. (2018) UCSF ChimeraX: Meeting modern challenges in visualization and analysis. *Protein Science* **27**, 14-25

12. Shornikov, A., Tran, H., Macias, J., Halavaty, A. S., Minasov, G., Anderson, W. F., and Kuhn, M. L. (2017) Structure of the *Bacillus anthracis* dTDP-L-rhamnose-biosynthetic enzyme dTDP-4-dehydrorhamnose 3,5-epimerase (RfbC). *Acta Crystallogr F Struct Biol Commun* **73**, 664-671

13. Christendat, D., Saridakis, V., Dharamsi, A., Bochkarev, A., Pai, E. F., Arrowsmith, C. H., and Edwards, A. M. (2000) Crystal structure of dTDP-4-keto-6-deoxy-D-hexulose 3,5-epimerase from *Methanobacterium thermoautotrophicum* complexed with dTDP. *J Biol Chem* **275**, 24608-24612

14. Giraud, M. F., Leonard, G. A., Field, R. A., Berlind, C., and Naismith, J. H. (2000) RmlC, the third enzyme of dTDP-L-rhamnose pathway, is a new class of epimerase. *Nat Struct Biol* **7**, 398-402

15. Jakimowicz, P., Tello, M., Meyers, C. L., Walsh, C. T., Buttner, M. J., Field, R. A., and Lawson, D. M. (2006) The 1.6-A resolution crystal structure of NovW: a 4-keto-6-deoxy sugar epimerase from the novobiocin biosynthetic gene cluster of *Streptomyces spheroides*. *Proteins* **63**, 261-265

16. Kubiak, R. L., Phillips, R. K., Zmudka, M. W., Ahn, M. R., Maka, E. M., Pyeatt, G. L., Roggensack, S. J., and Holden, H. M. (2012) Structural and functional studies on a 3'-epimerase involved in the biosynthesis of dTDP-6-deoxy-D-allose. *Biochemistry* **51**, 9375-9383

17. Kirkpatrick, P. N., Scaife, W., Hallis, T. M., Liu, H.-w., Spencer, J. B., and Williams, D. H. (2000) Characterisation of a sugar epimerase enzyme involved in the biosynthesis of a vancomycin-group antibiotic. *Chemical Communications*, 1565-1566

18. Stern, R. J., Lee, T. Y., Lee, T. J., Yan, W., Scherman, M. S., Vissa, V. D., Kim, S. K., Wanner, B. L., and McNeil, M. R. (1999) Conversion of dTDP-4-keto-6-deoxyglucose to free dTDP-4-keto-rhamnose by the rmIC gene products of *Escherichia coli* and *Mycobacterium tuberculosis*. *Microbiology (Reading)* **145 ( Pt 3)**, 663-671

19. York, W. S., Darvill, A. G., McNeil, M., Stevenson, T. T., and Albersheim, P. (1986) Isolation and characterization of plant cell walls and cell wall components. in *Methods in Enzymology*, Academic Press. pp 3-40
