# Supplementary material for: Spinning sugars in antigen biosynthesis: characterization of the Coxiella burnetii and Streptomyces griseus TDP-sugar epimerases
Source: J Biol Chem. 2022 Apr 6;298(5):101903. doi: 10.1016/j.jbc.2022.101903 (PMC9095892; doi:10.1016/j.jbc.2022.101903)
Supplement: Cross et al raw data [file mmc2.zip › ORE data drop/PDB deposition reports/D_1292118455_val-report-full_P1_1838_apo.pdf]

Full wwPDB X-ray Structure Validation Report ⓘ

Oct 5, 2021 – 11:55 am BST

PDB ID	:	7PVI
Title	:	dTDP-sugar epimerase
Deposited on	:	2021-10-04
Resolution	:	1.43 Å(reported)

This is a Full wwPDB X-ray Structure Validation Report.

This report is produced by the wwPDB biocuration pipeline after annotation of the structure.

We welcome your comments at *validation@mail.wwpdb.org*
A user guide is available at
https://www.wwpdb.org/validation/2017/XrayValidationReportHelp
with specific help available everywhere you see the ⓘ symbol.

CONFIDENTIAL VALIDATION REPORT

The following versions of software and data (see references ⓘ) were used in the production of this report:

MolProbity	:	4.02b-467
Mogul	:	1.8.5 (274361), CSD as541be (2020)
Xtriage (Phenix)	:	1.13
EDS	:	2.23.2
buster-report	:	1.1.7 (2018)
Percentile statistics	:	20191225.v01 (using entries in the PDB archive December 25th 2019)
Refmac	:	5.8.0267
CCP4	:	7.1.010 (Gargrove)
Ideal geometry (proteins)	:	Engh & Huber (2001)
Ideal geometry (DNA, RNA)	:	Parkinson et al. (1996)
Validation Pipeline (wwPDB-VP)	:	2.23.2

1 Overall quality at a glance ⓘ

The following experimental techniques were used to determine the structure:
X-RAY DIFFRACTION

The reported resolution of this entry is 1.43 Å.

Percentile scores (ranging between 0-100) for global validation metrics of the entry are shown in the following graphic. The table shows the number of entries on which the scores are based.

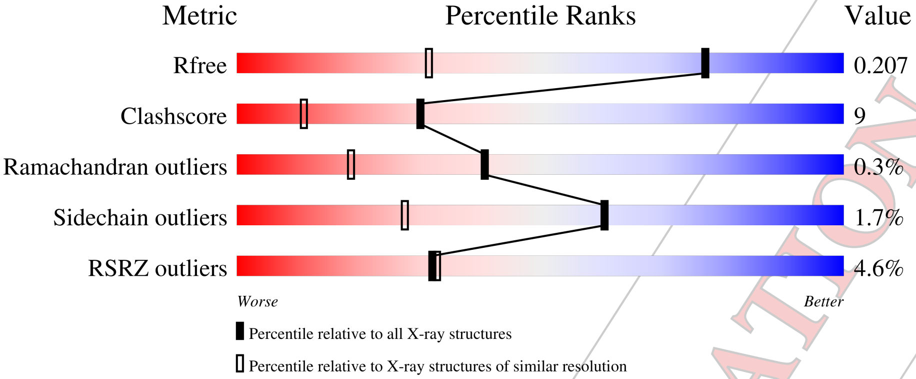

Metric	Whole archive (#Entries)	Similar resolution (#Entries, resolution range(Å))
R_{free}	130704	2021 (1.46-1.42)
Clashscore	141614	2086 (1.46-1.42)
Ramachandran outliers	138981	2047 (1.46-1.42)
Sidechain outliers	138945	2047 (1.46-1.42)
RSRZ outliers	127900	1993 (1.46-1.42)

The table below summarises the geometric issues observed across the polymeric chains and their fit to the electron density. The red, orange, yellow and green segments of the lower bar indicate the fraction of residues that contain outliers for >=3, 2, 1 and 0 types of geometric quality criteria respectively. A grey segment represents the fraction of residues that are not modelled. The numeric value for each fraction is indicated below the corresponding segment, with a dot representing fractions <=5% The upper red bar (where present) indicates the fraction of residues that have poor fit to the electron density. The numeric value is given above the bar.

Mol	Chain	Length	Quality of chain
1	AAA	211	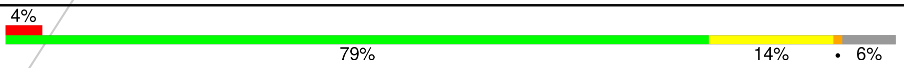
1	BBB	211	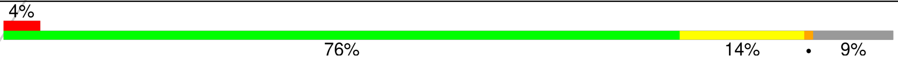

CONFIDENTIAL VALIDATION REPORT

2 Entry composition (i)

There are 6 unique types of molecules in this entry. The entry contains 4052 atoms, of which 0 are hydrogens and 0 are deuteriums.

In the tables below, the ZeroOcc column contains the number of atoms modelled with zero occupancy, the AltConf column contains the number of residues with at least one atom in alternate conformation and the Trace column contains the number of residues modelled with at most 2 atoms.

- Molecule 1 is a protein called dTDP-4-dehydrorhamnose 3,5-epimerase.

Mol	Chain	Residues	Atoms					ZeroOcc	AltConf	Trace
1	AAA	199	Total 1794	C 1180	N 297	O 308	S 9	0	29	0
1	BBB	191	Total 1746	C 1148	N 285	O 304	S 9	0	32	0

There are 44 discrepancies between the modelled and reference sequences:

Chain	Residue	Modelled	Actual	Comment	Reference
AAA	-21	MET	-	initiating methionine	UNP Q83AP3
AAA	-20	HIS	-	expression tag	UNP Q83AP3
AAA	-19	HIS	-	expression tag	UNP Q83AP3
AAA	-18	HIS	-	expression tag	UNP Q83AP3
AAA	-17	HIS	-	expression tag	UNP Q83AP3
AAA	-16	HIS	-	expression tag	UNP Q83AP3
AAA	-15	HIS	-	expression tag	UNP Q83AP3
AAA	-14	SER	-	expression tag	UNP Q83AP3
AAA	-13	SER	-	expression tag	UNP Q83AP3
AAA	-12	GLY	-	expression tag	UNP Q83AP3
AAA	-11	VAL	-	expression tag	UNP Q83AP3
AAA	-10	ASP	-	expression tag	UNP Q83AP3
AAA	-9	LEU	-	expression tag	UNP Q83AP3
AAA	-8	GLY	-	expression tag	UNP Q83AP3
AAA	-7	THR	-	expression tag	UNP Q83AP3
AAA	-6	GLU	-	expression tag	UNP Q83AP3
AAA	-5	ASN	-	expression tag	UNP Q83AP3
AAA	-4	LEU	-	expression tag	UNP Q83AP3
AAA	-3	TYR	-	expression tag	UNP Q83AP3
AAA	-2	PHE	-	expression tag	UNP Q83AP3
AAA	-1	GLN	-	expression tag	UNP Q83AP3
AAA	0	SER	-	expression tag	UNP Q83AP3
BBB	-21	MET	-	initiating methionine	UNP Q83AP3
BBB	-20	HIS	-	expression tag	UNP Q83AP3
BBB	-19	HIS	-	expression tag	UNP Q83AP3

Continued on next page...

CONFIDENTIAL VALIDATION REPORT

Continued from previous page...

Chain	Residue	Modelled	Actual	Comment	Reference
BBB	-18	HIS	-	expression tag	UNP Q83AP3
BBB	-17	HIS	-	expression tag	UNP Q83AP3
BBB	-16	HIS	-	expression tag	UNP Q83AP3
BBB	-15	HIS	-	expression tag	UNP Q83AP3
BBB	-14	SER	-	expression tag	UNP Q83AP3
BBB	-13	SER	-	expression tag	UNP Q83AP3
BBB	-12	GLY	-	expression tag	UNP Q83AP3
BBB	-11	VAL	-	expression tag	UNP Q83AP3
BBB	-10	ASP	-	expression tag	UNP Q83AP3
BBB	-9	LEU	-	expression tag	UNP Q83AP3
BBB	-8	GLY	-	expression tag	UNP Q83AP3
BBB	-7	THR	-	expression tag	UNP Q83AP3
BBB	-6	GLU	-	expression tag	UNP Q83AP3
BBB	-5	ASN	-	expression tag	UNP Q83AP3
BBB	-4	LEU	-	expression tag	UNP Q83AP3
BBB	-3	TYR	-	expression tag	UNP Q83AP3
BBB	-2	PHE	-	expression tag	UNP Q83AP3
BBB	-1	GLN	-	expression tag	UNP Q83AP3
BBB	0	SER	-	expression tag	UNP Q83AP3

- Molecule 2 is beta-D-xylopyranose (three-letter code: XYP) (formula: $C_5H_{10}O_5$) (labeled as "Ligand of Interest" by depositor).

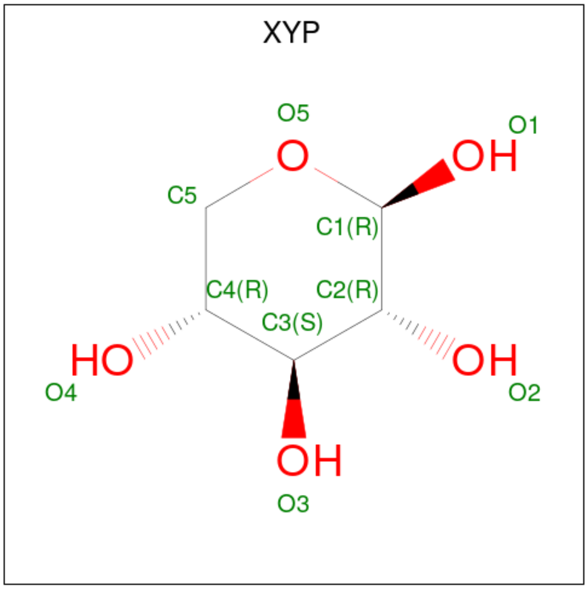

Mol	Chain	Residues	Atoms			ZeroOcc	AltConf
			Total	C	O		
2	AAA	1	10	5	5	0	0

Continued on next page...

CONFIDENTIAL VALIDATION REPORT

Continued from previous page...

Mol	Chain	Residues	Atoms	ZeroOcc	AltConf
2	BBB	1	Total C O<br>10 5 5	0	0

- Molecule 3 is alpha-D-xylopyranose (three-letter code: XYS) (formula: $C_5H_{10}O_5$) (labeled as "Ligand of Interest" by depositor).

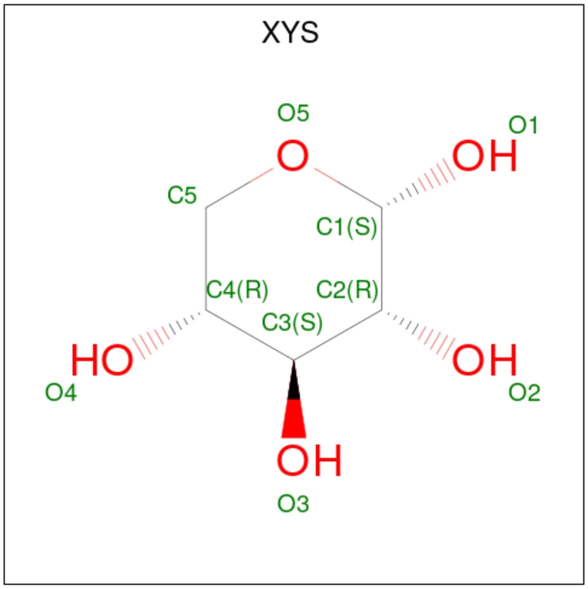

Mol	Chain	Residues	Atoms	ZeroOcc	AltConf
3	AAA	1	Total C O<br>10 5 5	0	0
3	BBB	1	Total C O<br>10 5 5	0	0

- Molecule 4 is SODIUM ION (three-letter code: NA) (formula: Na).

Mol	Chain	Residues	Atoms	ZeroOcc	AltConf
4	AAA	3	Total Na<br>3 3	0	0
4	BBB	3	Total Na<br>3 3	0	0

- Molecule 5 is CITRATE ANION (three-letter code: FLC) (formula: $C_6H_5O_7$).

CONFIDENTIAL VALIDATION REPORT

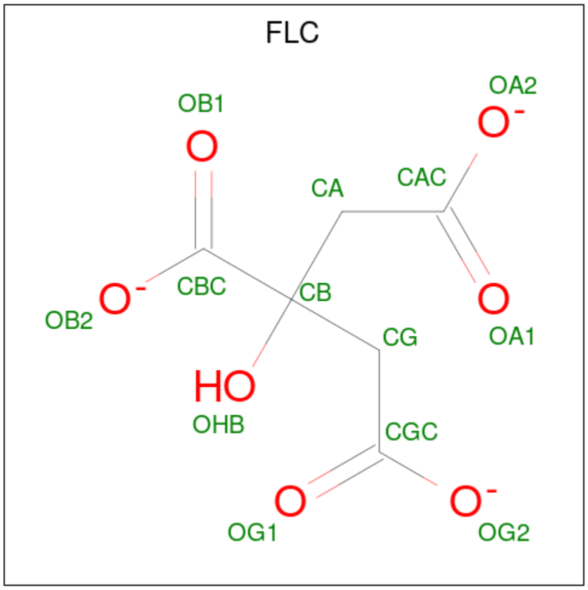

Mol	Chain	Residues	Atoms	ZeroOcc	AltConf
5	BBB	1	Total C O<br>13 6 7	0	0

- Molecule 6 is water.

Mol	Chain	Residues	Atoms	ZeroOcc	AltConf
6	AAA	226	Total O<br>226 226	0	0
6	BBB	227	Total O<br>227 227	0	0

CONFIDENTIAL VALIDATION REPORT

3 Residue-property plots (i)

These plots are drawn for all protein, RNA, DNA and oligosaccharide chains in the entry. The first graphic for a chain summarises the proportions of the various outlier classes displayed in the second graphic. The second graphic shows the sequence view annotated by issues in geometry and electron density. Residues are color-coded according to the number of geometric quality criteria for which they contain at least one outlier: green = 0, yellow = 1, orange = 2 and red = 3 or more. A red dot above a residue indicates a poor fit to the electron density (RSRZ > 2). Stretches of 2 or more consecutive residues without any outlier are shown as a green connector. Residues present in the sample, but not in the model, are shown in grey.

- Molecule 1: dTDP-4-dehydrorhamnose 3,5-epimerase

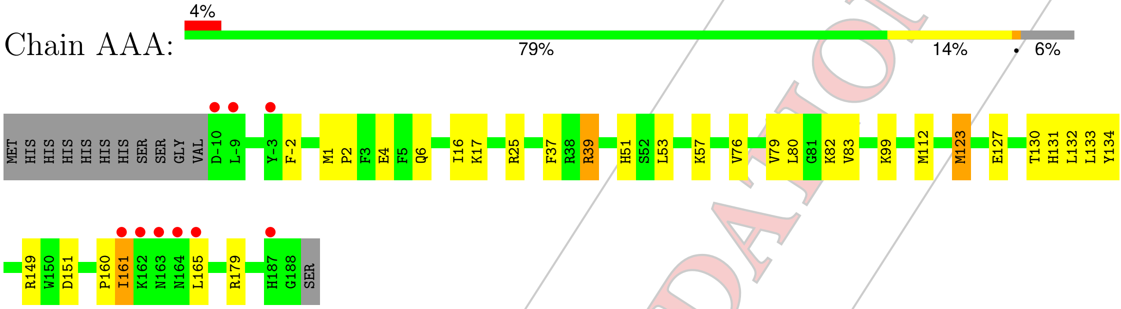

- Molecule 1: dTDP-4-dehydrorhamnose 3,5-epimerase

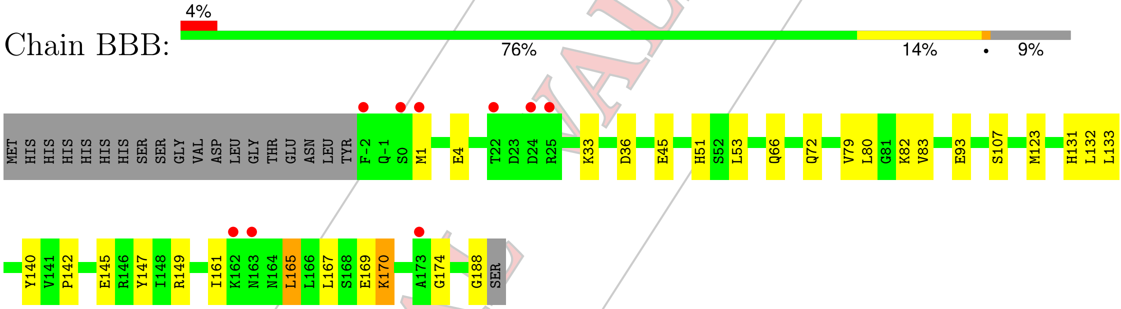

CONFIDENTIAL VALIDATION REPORT

4 Data and refinement statistics (i)

Property	Value	Source
Space group	C 1 2 1	Depositor
Cell constants<br>a, b, c, α, β, γ	77.03Å 84.54Å 77.67Å<br>90.00° 113.59° 90.00°	Depositor
Resolution (Å)	51.39 – 1.43<br>71.18 – 1.43	Depositor<br>EDS
% Data completeness<br>(in resolution range)	65.7 (51.39-1.43)<br>65.7 (71.18-1.43)	Depositor<br>EDS
R_{merge}	(Not available)	Depositor
R_{sym}	(Not available)	Depositor
$< I/\sigma(I) >$ [1]	1.13 (at 1.43Å)	Xtriage
Refinement program	REFMAC 5.8.0267	Depositor
R, R_{free}	0.167 , 0.199<br>0.172 , 0.207	Depositor<br>DCC
R_{free} test set	2739 reflections (4.99%)	wwPDB-VP
Wilson B-factor (Å^2)	18.5	Xtriage
Anisotropy	0.047	Xtriage
Bulk solvent k_{sol}(e/Å^3), B_{sol}(Å^2)	(Not available) , (Not available)	EDS
L-test for twinning[2]	$< \|L\| > = 0.49$, $< L^2 > = 0.32$	Xtriage
Estimated twinning fraction	No twinning to report.	Xtriage
F_o,F_c correlation	0.97	EDS
Total number of atoms	4052	wwPDB-VP
Average B, all atoms (Å^2)	23.0	wwPDB-VP

Xtriage's analysis on translational NCS is as follows: *The largest off-origin peak in the Patterson function is 6.21% of the height of the origin peak. No significant pseudotranslation is detected.*

[1] Intensities estimated from amplitudes.

[2] Theoretical values of $< |L| >$, $< L^2 >$ for acentric reflections are 0.5, 0.333 respectively for untwinned datasets, and 0.375, 0.2 for perfectly twinned datasets.

CONFIDENTIAL VALIDATION REPORT

5 Model quality (i)

5.1 Standard geometry (i)

Bond lengths and bond angles in the following residue types are not validated in this section: XYP, NA, FLC, XYS

The Z score for a bond length (or angle) is the number of standard deviations the observed value is removed from the expected value. A bond length (or angle) with $|Z| > 5$ is considered an outlier worth inspection. RMSZ is the root-mean-square of all Z scores of the bond lengths (or angles).

Mol	Chain	Bond lengths		Bond angles	
		RMSZ	#|Z| >5	RMSZ	#|Z| >5
1	AAA	0.58	0/1914	0.79	0/2581
1	BBB	0.59	0/1880	0.75	0/2534
All	All	0.58	0/3794	0.77	0/5115

There are no bond length outliers.

There are no bond angle outliers.

There are no chirality outliers.

There are no planarity outliers.

5.2 Too-close contacts (i)

In the following table, the Non-H and H(model) columns list the number of non-hydrogen atoms and hydrogen atoms in the chain respectively. The H(added) column lists the number of hydrogen atoms added and optimized by MolProbity. The Clashes column lists the number of clashes within the asymmetric unit, whereas Symm-Clashes lists symmetry-related clashes.

Mol	Chain	Non-H	H(model)	H(added)	Clashes	Symm-Clashes
1	AAA	1794	0	1889	34	1
1	BBB	1746	0	1828	36	0
2	AAA	10	0	0	0	0
2	BBB	10	0	0	0	0
3	AAA	10	0	10	0	0
3	BBB	10	0	10	0	0
4	AAA	3	0	0	0	0
4	BBB	3	0	0	0	0
5	BBB	13	0	5	0	0
6	AAA	226	0	0	13	0
6	BBB	227	0	0	15	0
All	All	4052	0	3742	68	1

CONFIDENTIAL VALIDATION REPORT

The all-atom clashscore is defined as the number of clashes found per 1000 atoms (including hydrogen atoms). The all-atom clashscore for this structure is 9.

All (68) close contacts within the same asymmetric unit are listed below, sorted by their clash magnitude.

Atom-1	Atom-2	Interatomic distance (Å)	Clash overlap (Å)
1:AAA:57[B]:LYS:HE2	6:AAA:427:HOH:O	1.24	1.31
1:BBB:66[B]:GLN:NE2	6:BBB:303:HOH:O	1.58	1.29
1:BBB:80[B]:LEU:HD12	6:BBB:444:HOH:O	1.19	1.28
1:BBB:80[B]:LEU:CD1	6:BBB:444:HOH:O	1.71	1.24
1:AAA:112[B]:MET:HE1	6:AAA:464:HOH:O	0.99	1.16
1:AAA:112[A]:MET:SD	6:AAA:449:HOH:O	2.14	1.05
1:BBB:80[B]:LEU:CG	6:BBB:378:HOH:O	2.11	0.99
1:BBB:1[B]:MET:HG2	6:BBB:310:HOH:O	1.61	0.99
1:BBB:80[B]:LEU:CD2	6:BBB:378:HOH:O	2.10	0.98
1:BBB:80[B]:LEU:HG	6:BBB:378:HOH:O	1.64	0.98
1:AAA:112[A]:MET:CG	6:AAA:449:HOH:O	2.12	0.97
1:BBB:66[A]:GLN:OE1	1:BBB:140[A]:TYR:CD1	2.19	0.96
1:BBB:80[B]:LEU:HD21	6:BBB:378:HOH:O	1.68	0.94
1:AAA:80[B]:LEU:HD12	6:AAA:465:HOH:O	1.72	0.88
1:AAA:112[A]:MET:HG2	6:AAA:449:HOH:O	1.74	0.87
1:AAA:99[B]:LYS:NZ	6:AAA:302:HOH:O	2.06	0.86
1:AAA:1[B]:MET:HG2	1:AAA:2:PRO:HD2	1.59	0.84
1:BBB:145[B]:GLU:OE1	6:BBB:304:HOH:O	2.00	0.80
1:BBB:66[A]:GLN:OE1	1:BBB:140[A]:TYR:CE1	2.36	0.79
1:BBB:45[B]:GLU:OE2	6:BBB:305:HOH:O	2.01	0.79
1:AAA:83:VAL:HG11	1:AAA:132[B]:LEU:HD11	1.66	0.78
1:BBB:140[B]:TYR:CZ	1:BBB:142:PRO:HG3	2.20	0.77
1:BBB:93:GLU:OE1	6:BBB:302:HOH:O	2.05	0.73
1:AAA:83:VAL:CG1	1:AAA:132[B]:LEU:HD11	2.19	0.72
1:BBB:83:VAL:HG11	1:BBB:132[A]:LEU:HD21	1.76	0.68
1:AAA:80[B]:LEU:CD1	6:AAA:465:HOH:O	2.35	0.68
1:AAA:161[B]:ILE:HA	6:AAA:359:HOH:O	1.94	0.68
1:AAA:1[B]:MET:HG2	1:AAA:2:PRO:CD	2.26	0.64
1:BBB:66[A]:GLN:OE1	1:BBB:140[A]:TYR:HD1	1.77	0.63
1:BBB:169[B]:GLU:HG3	6:BBB:348:HOH:O	1.99	0.63
1:AAA:160[B]:PRO:O	1:AAA:161[B]:ILE:HB	1.98	0.62
1:BBB:165:LEU:HD23	1:BBB:167:LEU:HG	1.80	0.62
1:BBB:165:LEU:CD2	1:BBB:167:LEU:HG	2.30	0.62
1:BBB:1[B]:MET:SD	1:BBB:4[B]:GLU:HG2	2.42	0.60
1:BBB:80[B]:LEU:HD11	6:BBB:444:HOH:O	1.63	0.58
1:AAA:161[A]:ILE:HA	6:AAA:359:HOH:O	2.03	0.57
1:BBB:140[B]:TYR:CE2	1:BBB:142:PRO:HG3	2.39	0.56

Continued on next page...

CONFIDENTIAL VALIDATION REPORT

Continued from previous page...

Atom-1	Atom-2	Interatomic distance (Å)	Clash overlap (Å)
1:AAA:82[B]:LYS:HE2	1:AAA:127:GLU:HG3	1.88	0.55
1:BBB:149:ARG:HG2	1:BBB:174:GLY:O	2.10	0.51
1:BBB:66[B]:GLN:HE21	1:BBB:72:GLN:H	1.58	0.50
1:BBB:188:GLY:HA3	6:BBB:376:HOH:O	2.11	0.50
1:BBB:66[B]:GLN:NE2	1:BBB:72:GLN:H	2.09	0.50
1:BBB:161:ILE:HG12	1:BBB:165:LEU:HD12	1.95	0.48
1:AAA:76:VAL:CG1	1:AAA:132[A]:LEU:HD11	2.44	0.48
1:BBB:82[B]:LYS:NZ	6:BBB:306:HOH:O	2.32	0.47
1:BBB:79[B]:VAL:HG11	1:BBB:133:LEU:HD11	1.95	0.47
1:AAA:1[B]:MET:CG	1:AAA:2:PRO:HD2	2.39	0.47
1:AAA:132[A]:LEU:HD23	1:AAA:134:TYR:CZ	2.50	0.47
1:AAA:161[B]:ILE:CG2	1:AAA:165:LEU:HD21	2.46	0.45
1:AAA:53[A]:LEU:HD23	1:AAA:131:HIS:CD2	2.50	0.45
1:AAA:132[A]:LEU:HD23	1:AAA:134:TYR:OH	2.16	0.45
1:AAA:79[B]:VAL:HG11	1:AAA:133:LEU:HD11	1.98	0.45
1:BBB:83:VAL:CG1	1:BBB:132[A]:LEU:HD21	2.46	0.45
1:AAA:112[B]:MET:CE	6:AAA:464:HOH:O	1.87	0.44
1:BBB:147:TYR:CE2	1:BBB:170[A]:LYS:HG2	2.52	0.44
1:AAA:4[B]:GLU:OE1	1:AAA:17[B]:LYS:HE2	2.18	0.43
1:AAA:79[A]:VAL:HG23	1:BBB:51:HIS:CE1	2.53	0.43
1:AAA:83:VAL:HG13	1:AAA:132[B]:LEU:HD11	1.98	0.42
1:BBB:33[B]:LYS:HD2	1:BBB:36[B]:ASP:OD1	2.20	0.42
1:BBB:53[B]:LEU:HD23	1:BBB:131:HIS:CD2	2.54	0.41
1:AAA:149:ARG:HG3	1:AAA:151:ASP:OD1	2.20	0.41
1:AAA:51:HIS:CE1	1:BBB:79[A]:VAL:HG23	2.55	0.41
1:AAA:123:MET:HE2	1:AAA:132[B]:LEU:HD13	2.03	0.41
1:AAA:130:THR:HG22	1:AAA:132[B]:LEU:HD12	2.02	0.41
1:BBB:80[B]:LEU:HA	1:BBB:107:SER:HB3	2.01	0.41
1:AAA:16[B]:ILE:HD13	1:AAA:37:PHE:CE2	2.56	0.40
1:AAA:179:ARG:NH2	6:AAA:320:HOH:O	2.52	0.40
1:AAA:39[A]:ARG:NH1	6:AAA:326:HOH:O	2.55	0.40

All (1) symmetry-related close contacts are listed below. The label for Atom-2 includes the symmetry operator and encoded unit-cell translations to be applied.

Atom-1	Atom-2	Interatomic distance (Å)	Clash overlap (Å)
1:AAA:-2:PHE:CZ	1:AAA:6[A]:GLN:NE2[2_555]	2.18	0.02

CONFIDENTIAL VALIDATION REPORT

5.3 Torsion angles (i)

5.3.1 Protein backbone (i)

In the following table, the Percentiles column shows the percent Ramachandran outliers of the chain as a percentile score with respect to all X-ray entries followed by that with respect to entries of similar resolution.

The Analysed column shows the number of residues for which the backbone conformation was analysed, and the total number of residues.

Mol	Chain	Analysed	Favoured	Allowed	Outliers	Percentiles	
1	AAA	226/211 (107%)	224 (99%)	0	2 (1%)	17	3
1	BBB	221/211 (105%)	218 (99%)	3 (1%)	0	100	100
All	All	447/422 (106%)	442 (99%)	3 (1%)	2 (0%)	41	13

All (2) Ramachandran outliers are listed below:

Mol	Chain	Res	Type
1	AAA	161[A]	ILE
1	AAA	161[B]	ILE

5.3.2 Protein sidechains (i)

In the following table, the Percentiles column shows the percent sidechain outliers of the chain as a percentile score with respect to all X-ray entries followed by that with respect to entries of similar resolution.

The Analysed column shows the number of residues for which the sidechain conformation was analysed, and the total number of residues.

Mol	Chain	Analysed	Rotameric	Outliers	Percentiles	
1	AAA	206/189 (109%)	202 (98%)	4 (2%)	57	22
1	BBB	202/189 (107%)	197 (98%)	5 (2%)	47	13
All	All	408/378 (108%)	399 (98%)	9 (2%)	60	17

All (9) residues with a non-rotameric sidechain are listed below:

Mol	Chain	Res	Type
1	AAA	25	ARG
1	AAA	39[A]	ARG
1	AAA	39[B]	ARG
1	AAA	123	MET

Continued on next page...

CONFIDENTIAL VALIDATION REPORT

Continued from previous page...

Mol	Chain	Res	Type
1	BBB	123[A]	MET
1	BBB	123[B]	MET
1	BBB	165	LEU
1	BBB	170[A]	LYS
1	BBB	170[B]	LYS

Sometimes sidechains can be flipped to improve hydrogen bonding and reduce clashes. There are no such sidechains identified.

5.3.3 RNA (i)

There are no RNA molecules in this entry.

5.4 Non-standard residues in protein, DNA, RNA chains (i)

There are no non-standard protein/DNA/RNA residues in this entry.

5.5 Carbohydrates (i)

There are no monosaccharides in this entry.

5.6 Ligand geometry (i)

Of 11 ligands modelled in this entry, 6 are monoatomic - leaving 5 for Mogul analysis.

In the following table, the Counts columns list the number of bonds (or angles) for which Mogul statistics could be retrieved, the number of bonds (or angles) that are observed in the model and the number of bonds (or angles) that are defined in the Chemical Component Dictionary. The Link column lists molecule types, if any, to which the group is linked. The Z score for a bond length (or angle) is the number of standard deviations the observed value is removed from the expected value. A bond length (or angle) with $|Z| > 2$ is considered an outlier worth inspection. RMSZ is the root-mean-square of all Z scores of the bond lengths (or angles).

Mol	Type	Chain	Res	Link	Bond lengths			Bond angles		
					Counts	RMSZ	$\#\|Z\| > 2$	Counts	RMSZ	$\#\|Z\| > 2$
2	XYP	BBB	201	4	10,10,10	0.64	0	14,14,14	0.52	0
3	XYS	AAA	202	-	10,10,10	0.47	0	14,14,14	0.58	0
2	XYP	AAA	201	-	10,10,10	0.09	0	14,14,14	0.56	0
3	XYS	BBB	202	-	10,10,10	0.66	0	14,14,14	0.93	1 (7%)
5	FLC	BBB	203	-	3,12,12	0.89	0	3,17,17	1.01	0

CONFIDENTIAL VALIDATION REPORT

In the following table, the Chirals column lists the number of chiral outliers, the number of chiral centers analysed, the number of these observed in the model and the number defined in the Chemical Component Dictionary. Similar counts are reported in the Torsion and Rings columns. '-' means no outliers of that kind were identified.

Mol	Type	Chain	Res	Link	Chirals	Torsions	Rings
2	XYP	BBB	201	4	-	-	0/1/1/1
3	XYS	AAA	202	-	-	-	0/1/1/1
2	XYP	AAA	201	-	-	-	0/1/1/1
3	XYS	BBB	202	-	-	-	0/1/1/1
5	FLC	BBB	203	-	-	0/6/16/16	-

There are no bond length outliers.

All (1) bond angle outliers are listed below:

Mol	Chain	Res	Type	Atoms	Z	Observed(^{o})	Ideal(^{o})
3	BBB	202	XYS	C1-C2-C3	2.32	115.12	110.31

There are no chirality outliers.

There are no torsion outliers.

There are no ring outliers.

No monomer is involved in short contacts.

The following is a two-dimensional graphical depiction of Mogul quality analysis of bond lengths, bond angles, torsion angles, and ring geometry for all instances of the Ligand of Interest. In addition, ligands with molecular weight > 250 and outliers as shown on the validation Tables will also be included. For torsion angles, if less then 5% of the Mogul distribution of torsion angles is within 10 degrees of the torsion angle in question, then that torsion angle is considered an outlier. Any bond that is central to one or more torsion angles identified as an outlier by Mogul will be highlighted in the graph. For rings, the root-mean-square deviation (RMSD) between the ring in question and similar rings identified by Mogul is calculated over all ring torsion angles. If the average RMSD is greater than 60 degrees and the minimal RMSD between the ring in question and any Mogul-identified rings is also greater than 60 degrees, then that ring is considered an outlier. The outliers are highlighted in purple. The color gray indicates Mogul did not find sufficient equivalents in the CSD to analyse the geometry.

CONFIDENTIAL VALIDATION REPORT

Ligand XYP BBB 201

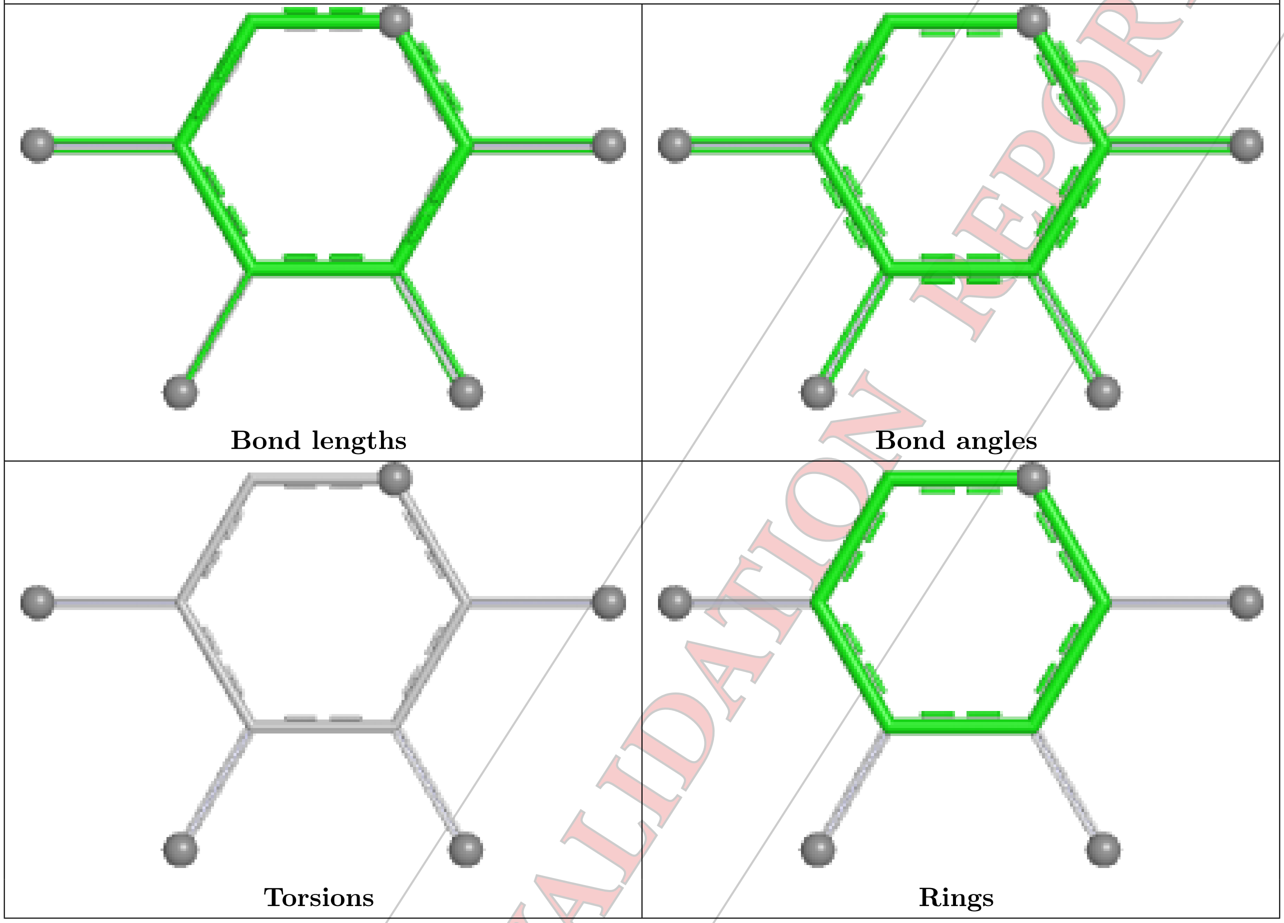

CONFIDENTIAL VALIDATION REPORT

Ligand XYS AAA 202

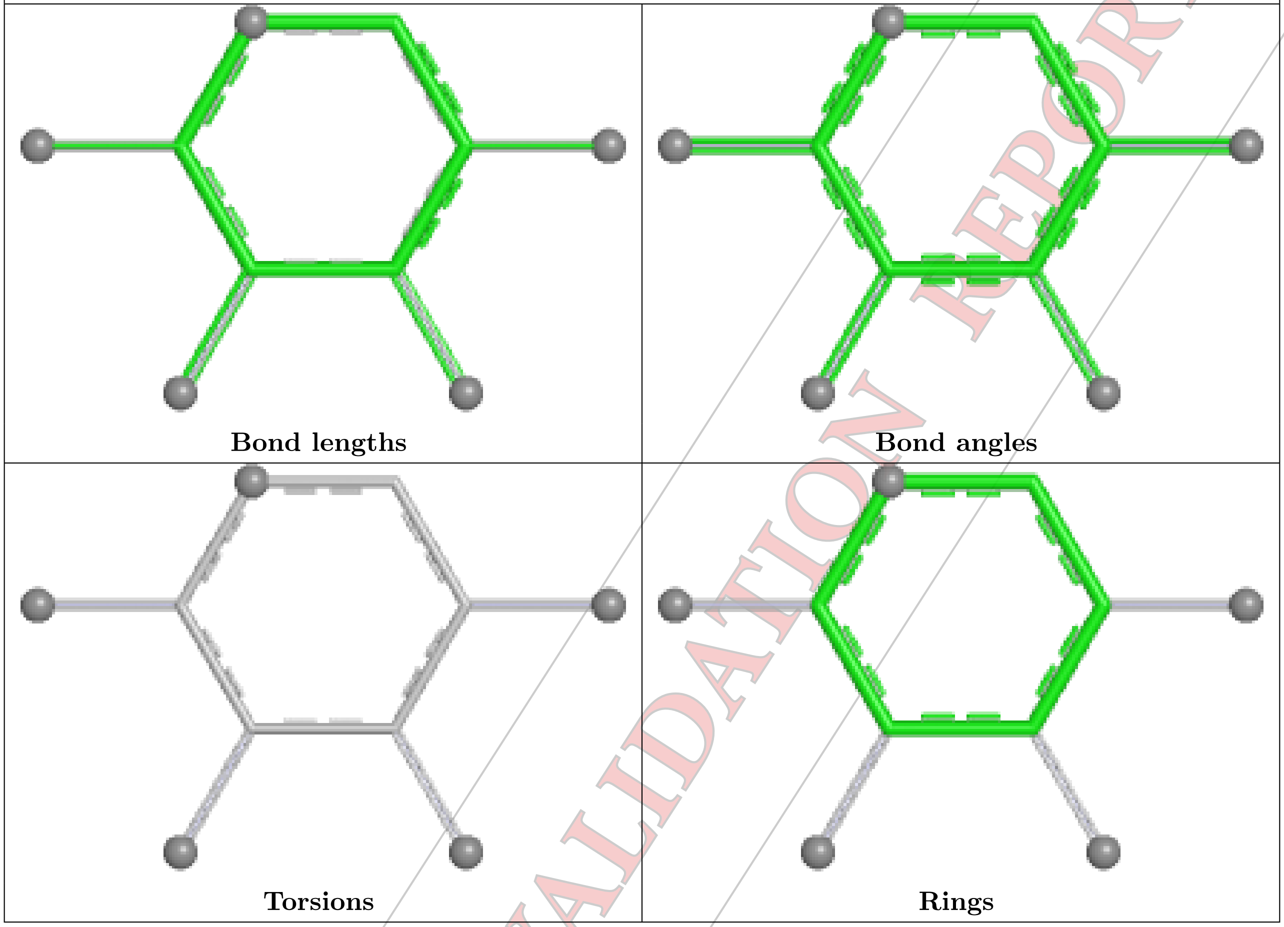

CONFIDENTIAL VALIDATION REPORT

Ligand XYP AAA 201

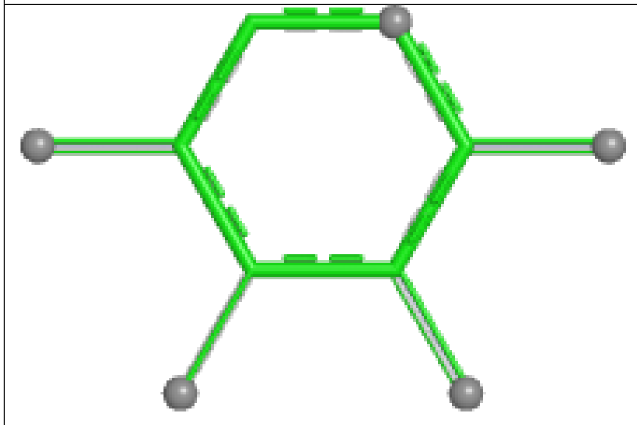

Bond lengths

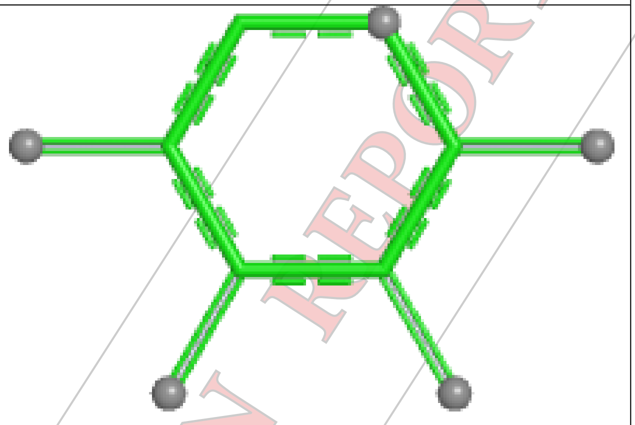

Bond angles

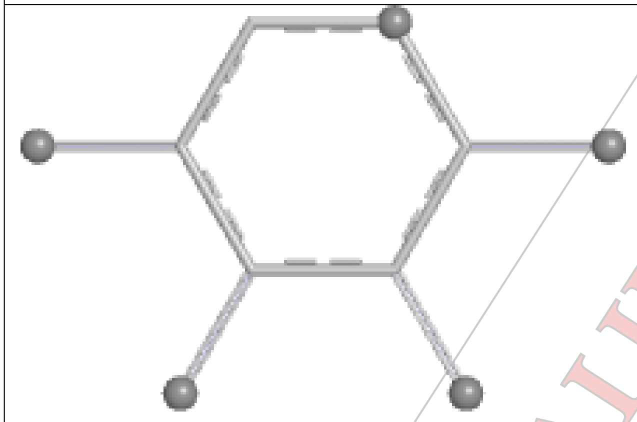

Torsions

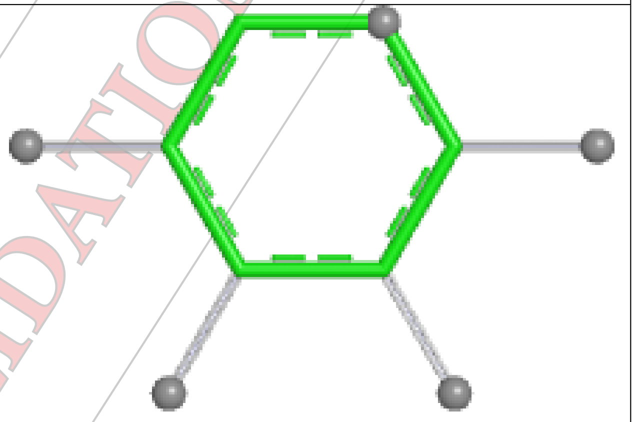

Rings

CONFIDENTIAL VALIDATION REPORT

Ligand XYS BBB 202

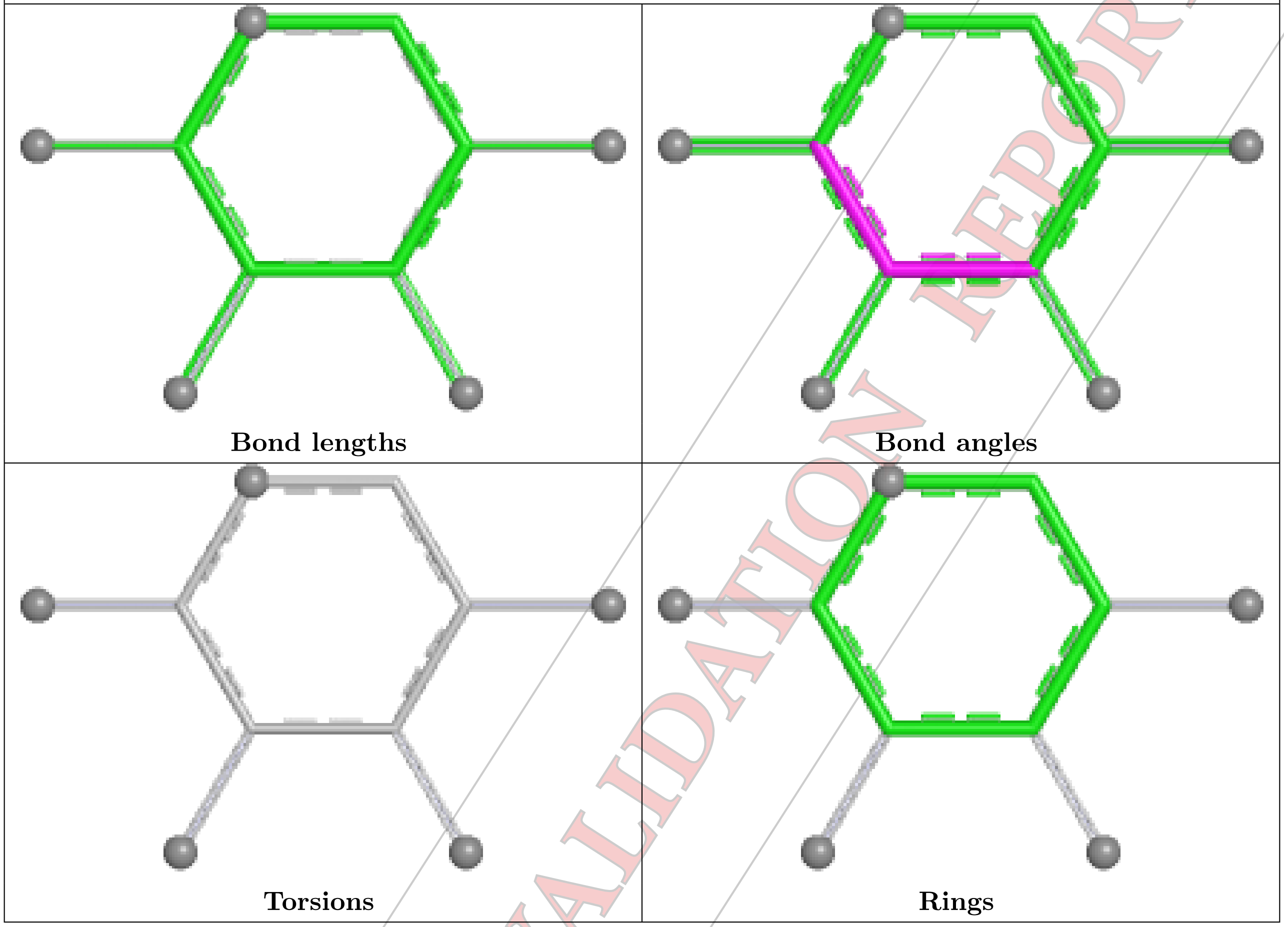

5.7 Other polymers (i)

There are no such residues in this entry.

5.8 Polymer linkage issues (i)

There are no chain breaks in this entry.

CONFIDENTIAL VALIDATION REPORT

6 Fit of model and data (i)

6.1 Protein, DNA and RNA chains (i)

In the following table, the column labelled '#RSRZ> 2' contains the number (and percentage) of RSRZ outliers, followed by percent RSRZ outliers for the chain as percentile scores relative to all X-ray entries and entries of similar resolution. The OWAB column contains the minimum, median, 95th percentile and maximum values of the occupancy-weighted average B-factor per residue. The column labelled 'Q< 0.9' lists the number of (and percentage) of residues with an average occupancy less than 0.9.

Mol	Chain	Analysed	<RSRZ>	#RSRZ>2			OWAB(Å^2)	Q<0.9
1	AAA	199/211 (94%)	0.30	9 (4%)	33	34	12, 20, 39, 64	0
1	BBB	191/211 (90%)	0.20	9 (4%)	31	32	11, 18, 44, 77	0
All	All	390/422 (92%)	0.25	18 (4%)	32	33	11, 18, 41, 77	0

All (18) RSRZ outliers are listed below:

Mol	Chain	Res	Type	RSRZ
1	BBB	-2	PHE	5.3
1	AAA	-9[A]	LEU	5.1
1	BBB	1[A]	MET	4.8
1	AAA	161[A]	ILE	4.5
1	AAA	-3	TYR	4.3
1	BBB	0	SER	4.3
1	AAA	163[A]	ASN	3.7
1	AAA	162	LYS	3.6
1	BBB	24[A]	ASP	3.4
1	AAA	164	ASN	3.2
1	BBB	162	LYS	2.5
1	BBB	163	ASN	2.5
1	AAA	165	LEU	2.5
1	AAA	-10	ASP	2.4
1	AAA	187	HIS	2.3
1	BBB	173	ALA	2.3
1	BBB	25	ARG	2.2
1	BBB	22	THR	2.0

6.2 Non-standard residues in protein, DNA, RNA chains (i)

There are no non-standard protein/DNA/RNA residues in this entry.

CONFIDENTIAL VALIDATION REPORT

6.3 Carbohydrates (i)

There are no monosaccharides in this entry.

6.4 Ligands (i)

In the following table, the Atoms column lists the number of modelled atoms in the group and the number defined in the chemical component dictionary. The B-factors column lists the minimum, median, 95th percentile and maximum values of B factors of atoms in the group. The column labelled 'Q< 0.9' lists the number of atoms with occupancy less than 0.9.

Mol	Type	Chain	Res	Atoms	RSCC	RSR	B-factors(Å^2)	Q<0.9
3	XYS	AAA	202	10/10	0.71	0.22	50,52,53,54	10
4	NA	AAA	204	1/1	0.85	0.66	53,53,53,53	0
4	NA	AAA	205	1/1	0.88	0.09	40,40,40,40	0
4	NA	BBB	204	1/1	0.90	0.13	44,44,44,44	0
2	XYP	AAA	201	10/10	0.92	0.12	20,22,23,24	10
2	XYP	BBB	201	10/10	0.92	0.13	20,24,27,29	0
3	XYS	BBB	202	10/10	0.93	0.14	27,30,34,35	0
5	FLC	BBB	203	13/13	0.94	0.12	20,21,23,25	0
4	NA	BBB	206	1/1	0.96	0.23	21,21,21,21	1
4	NA	BBB	205	1/1	0.98	0.11	38,38,38,38	0
4	NA	AAA	203	1/1	0.99	0.23	38,38,38,38	0

The following is a graphical depiction of the model fit to experimental electron density of all instances of the Ligand of Interest. In addition, ligands with molecular weight > 250 and outliers as shown on the geometry validation Tables will also be included. Each fit is shown from different orientation to approximate a three-dimensional view.

CONFIDENTIAL VALIDATION REPORT

Electron density around XYS AAA 202:

2mF$_o$-DF$_c$ (at 0.7 rmsd) in gray
mF$_o$-DF$_c$ (at 3 rmsd) in purple (negative)
and green (positive)

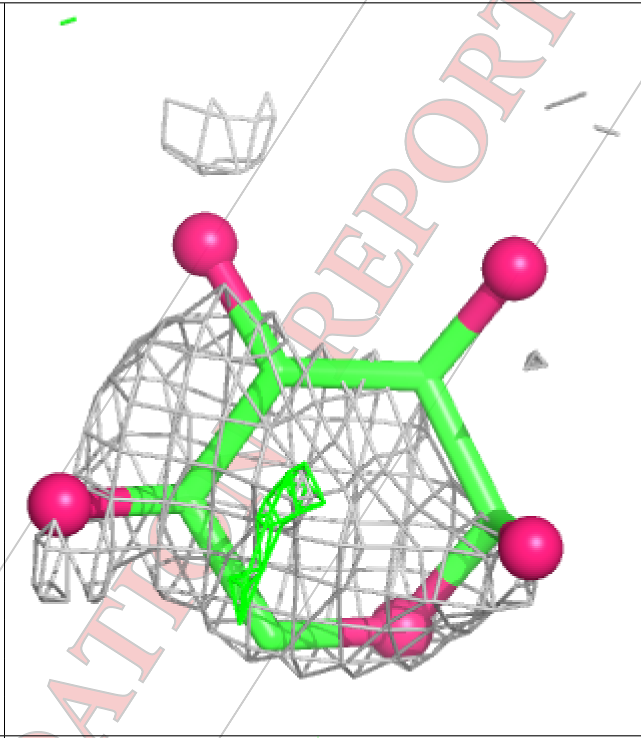

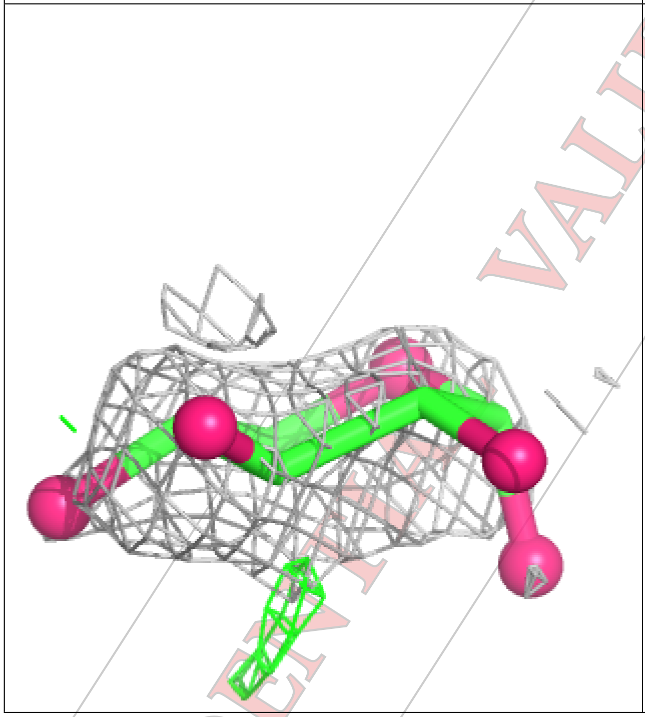

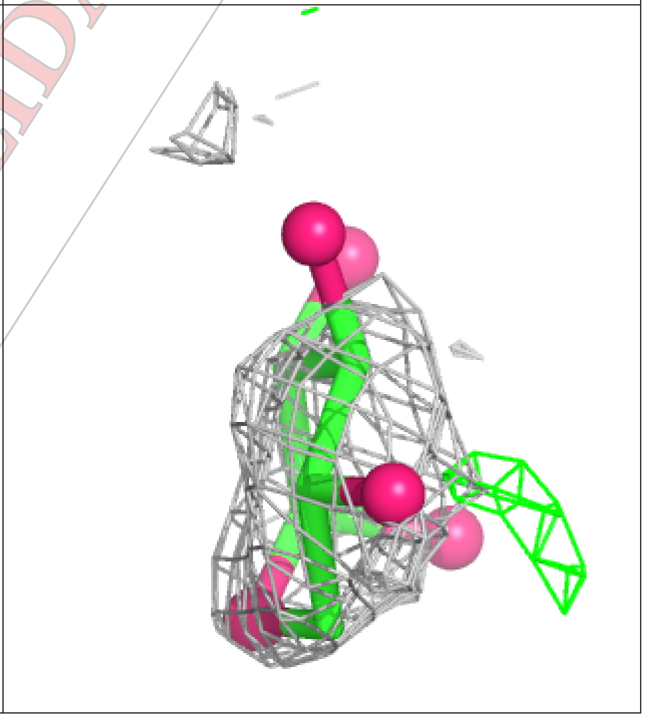

CONFIDENTIAL VALIDATION REPORT

Electron density around XYP AAA 201:

$2mF_o$-DF_c (at 0.7 rmsd) in gray
mF_o-DF_c (at 3 rmsd) in purple (negative)
and green (positive)

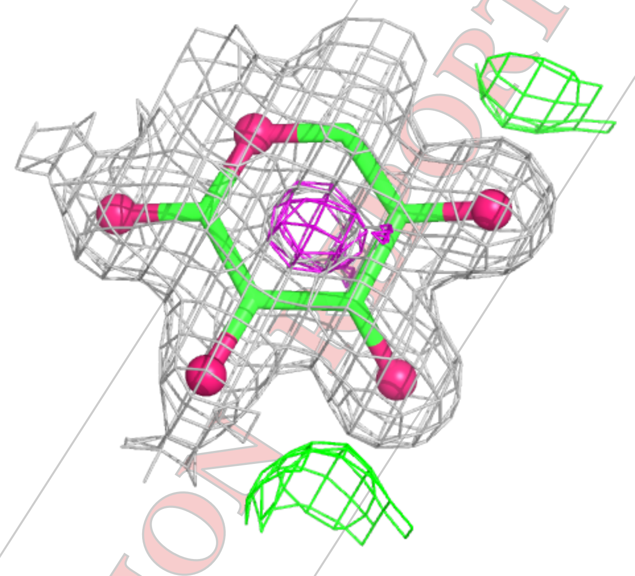

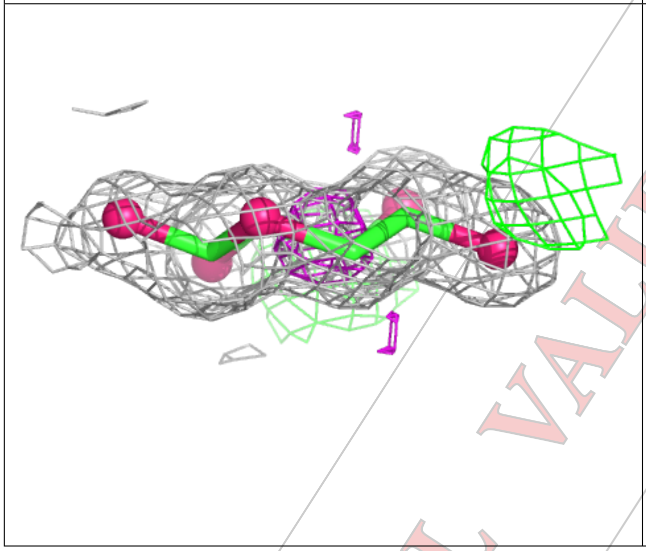

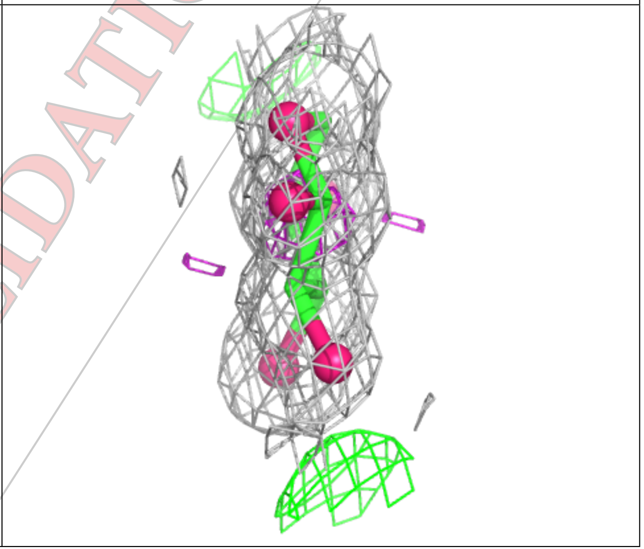

CONFIDENTIAL VALIDATION REPORT

Electron density around XYP BBB 201:

$2mF_o$-DF_c (at 0.7 rmsd) in gray
mF_o-DF_c (at 3 rmsd) in purple (negative)
and green (positive)

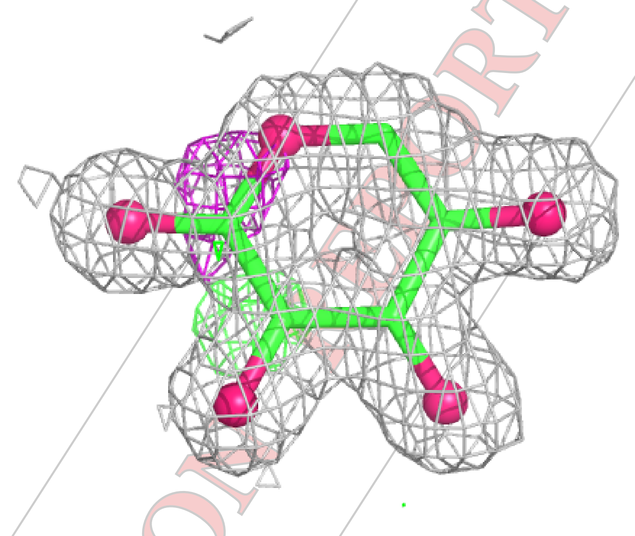

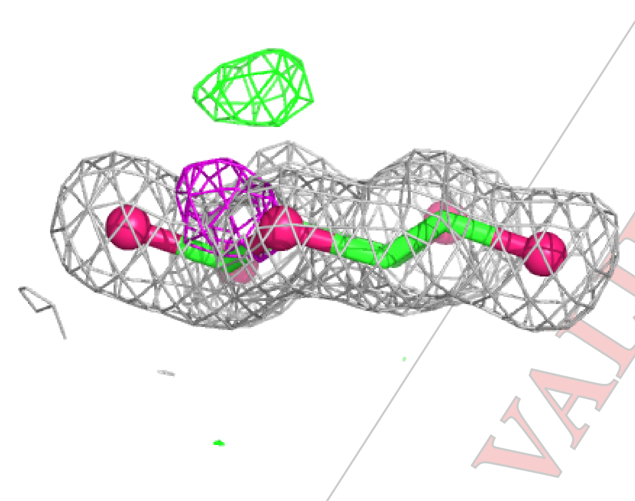

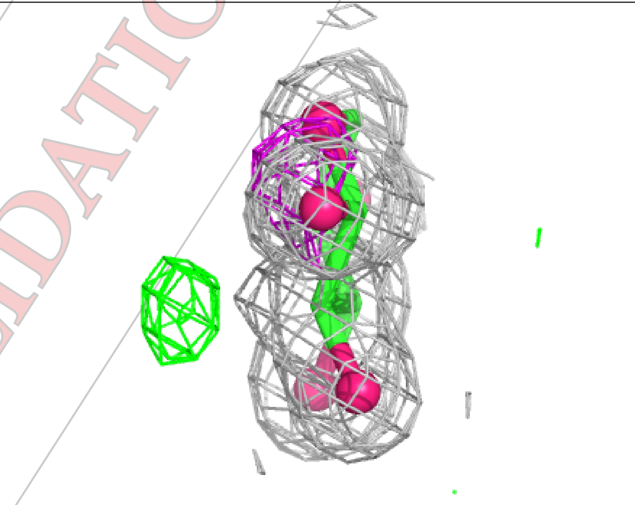

CONFIDENTIAL VALIDATION REPORT

Electron density around XYS BBB 202:

2mF$_o$-DF$_c$ (at 0.7 rmsd) in gray
mF$_o$-DF$_c$ (at 3 rmsd) in purple (negative)
and green (positive)

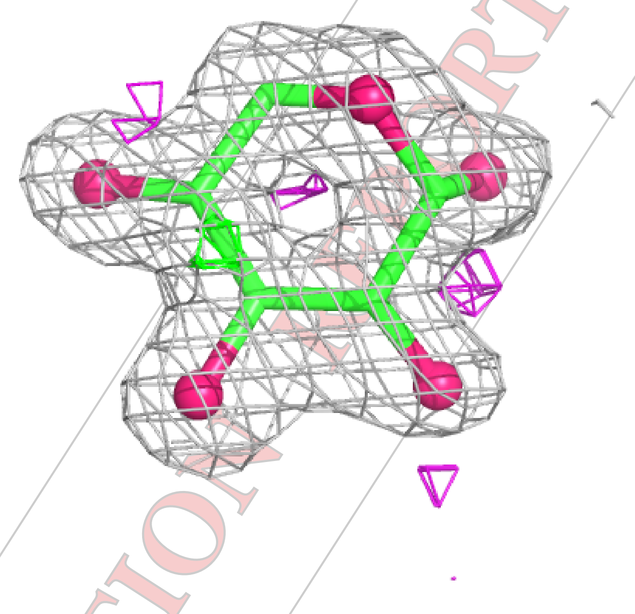

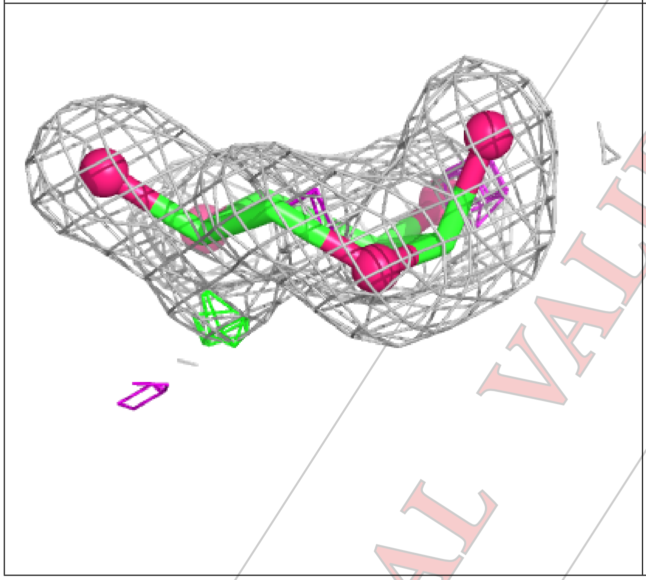

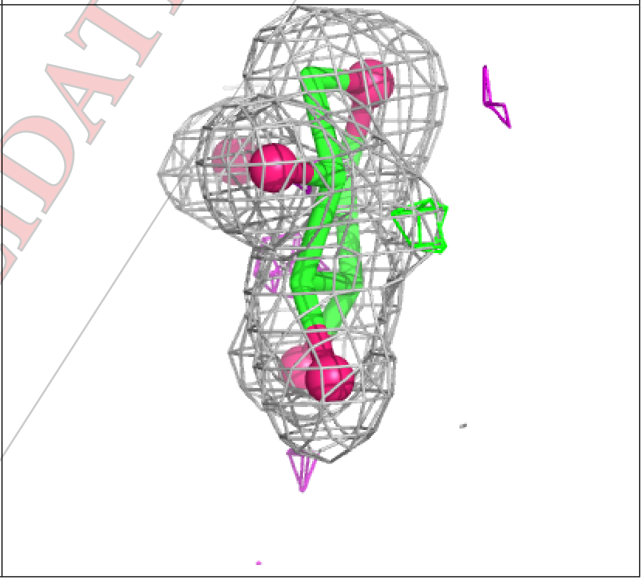

6.5 Other polymers ⓘ

There are no such residues in this entry.

CONFIDENTIAL VALIDATION REPORT
