# Supplementary material for: Spinning sugars in antigen biosynthesis: characterization of the Coxiella burnetii and Streptomyces griseus TDP-sugar epimerases
Source: J Biol Chem. 2022 Apr 6;298(5):101903. doi: 10.1016/j.jbc.2022.101903 (PMC9095892; doi:10.1016/j.jbc.2022.101903)
Supplement: Cross et al raw data [file mmc2.zip › ORE data drop/PDB deposition reports/D_1292118519_val-report-full_P1_1838_TDP.pdf]

Full wwPDB X-ray Structure Validation Report (i)

Oct 8, 2021 – 09:17 am BST

PDB ID	:	7PWB
Title	:	dTDP-sugar epimerase from Coxiella burnetii in complex with dTDP
Deposited on	:	2021-10-06
Resolution	:	1.87 Å(reported)

This is a Full wwPDB X-ray Structure Validation Report.

This report is produced by the wwPDB biocuration pipeline after annotation of the structure.

We welcome your comments at *validation@mail.wwpdb.org*
A user guide is available at
https://www.wwpdb.org/validation/2017/XrayValidationReportHelp
with specific help available everywhere you see the (i) symbol.

CONFIDENTIAL VALIDATION REPORT

The following versions of software and data (see references (i)) were used in the production of this report:

MolProbity	:	4.02b-467
Mogul	:	1.8.5 (274361), CSD as541be (2020)
Xtriage (Phenix)	:	1.13
EDS	:	2.23.2
buster-report	:	1.1.7 (2018)
Percentile statistics	:	20191225.v01 (using entries in the PDB archive December 25th 2019)
Refmac	:	5.8.0267
CCP4	:	7.1.010 (Gargrove)
Ideal geometry (proteins)	:	Engh & Huber (2001)
Ideal geometry (DNA, RNA)	:	Parkinson et al. (1996)
Validation Pipeline (wwPDB-VP)	:	2.23.2

1 Overall quality at a glance (i)

The following experimental techniques were used to determine the structure:
X-RAY DIFFRACTION

The reported resolution of this entry is 1.87 Å.

Percentile scores (ranging between 0-100) for global validation metrics of the entry are shown in the following graphic. The table shows the number of entries on which the scores are based.

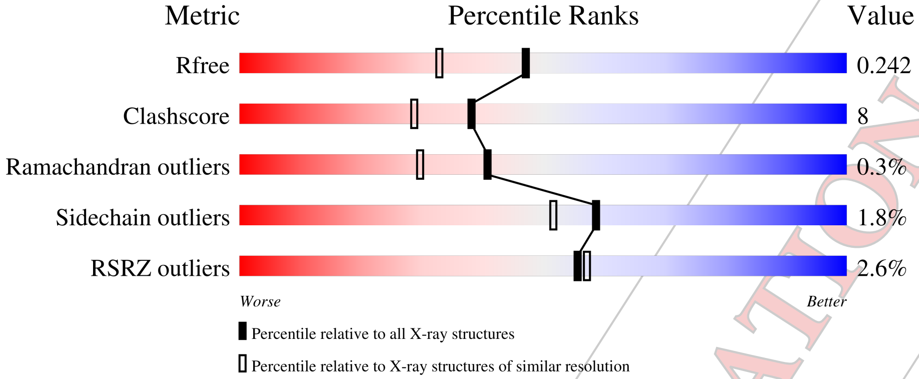

Metric	Whole archive (#Entries)	Similar resolution (#Entries, resolution range(Å))
R_{free}	130704	9470 (1.90-1.86)
Clashscore	141614	10282 (1.90-1.86)
Ramachandran outliers	138981	10152 (1.90-1.86)
Sidechain outliers	138945	10152 (1.90-1.86)
RSRZ outliers	127900	9303 (1.90-1.86)

The table below summarises the geometric issues observed across the polymeric chains and their fit to the electron density. The red, orange, yellow and green segments of the lower bar indicate the fraction of residues that contain outliers for >=3, 2, 1 and 0 types of geometric quality criteria respectively. A grey segment represents the fraction of residues that are not modelled. The numeric value for each fraction is indicated below the corresponding segment, with a dot representing fractions <=5% The upper red bar (where present) indicates the fraction of residues that have poor fit to the electron density. The numeric value is given above the bar.

Mol	Chain	Length	Quality of chain
1	AAA	211	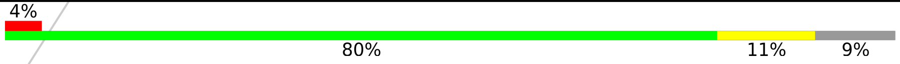
1	BBB	211	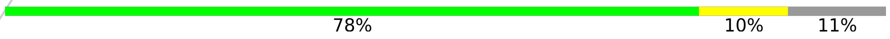

The following table lists non-polymeric compounds, carbohydrate monomers and non-standard residues in protein, DNA, RNA chains that are outliers for geometric or electron-density-fit criteria:

CONFIDENTIAL VALIDATION REPORT

Mol	Type	Chain	Res	Chirality	Geometry	Clashes	Electron density
4	EDO	AAA	205	-	-	X	-

CONFIDENTIAL VALIDATION REPORT

2 Entry composition (i)

There are 5 unique types of molecules in this entry. The entry contains 3766 atoms, of which 0 are hydrogens and 0 are deuteriums.

In the tables below, the ZeroOcc column contains the number of atoms modelled with zero occupancy, the AltConf column contains the number of residues with at least one atom in alternate conformation and the Trace column contains the number of residues modelled with at most 2 atoms.

- Molecule 1 is a protein called dTDP-4-dehydrorhamnose 3,5-epimerase.

Mol	Chain	Residues	Atoms					ZeroOcc	AltConf	Trace
1	AAA	193	Total 1707	C 1119	N 286	O 293	S 9	0	20	0
1	BBB	187	Total 1617	C 1052	N 278	O 281	S 6	0	14	0

There are 44 discrepancies between the modelled and reference sequences:

Chain	Residue	Modelled	Actual	Comment	Reference
AAA	-21	MET	-	initiating methionine	UNP Q83AP3
AAA	-20	HIS	-	expression tag	UNP Q83AP3
AAA	-19	HIS	-	expression tag	UNP Q83AP3
AAA	-18	HIS	-	expression tag	UNP Q83AP3
AAA	-17	HIS	-	expression tag	UNP Q83AP3
AAA	-16	HIS	-	expression tag	UNP Q83AP3
AAA	-15	HIS	-	expression tag	UNP Q83AP3
AAA	-14	SER	-	expression tag	UNP Q83AP3
AAA	-13	SER	-	expression tag	UNP Q83AP3
AAA	-12	GLY	-	expression tag	UNP Q83AP3
AAA	-11	VAL	-	expression tag	UNP Q83AP3
AAA	-10	ASP	-	expression tag	UNP Q83AP3
AAA	-9	LEU	-	expression tag	UNP Q83AP3
AAA	-8	GLY	-	expression tag	UNP Q83AP3
AAA	-7	THR	-	expression tag	UNP Q83AP3
AAA	-6	GLU	-	expression tag	UNP Q83AP3
AAA	-5	ASN	-	expression tag	UNP Q83AP3
AAA	-4	LEU	-	expression tag	UNP Q83AP3
AAA	-3	TYR	-	expression tag	UNP Q83AP3
AAA	-2	PHE	-	expression tag	UNP Q83AP3
AAA	-1	GLN	-	expression tag	UNP Q83AP3
AAA	0	SER	-	expression tag	UNP Q83AP3
BBB	-21	MET	-	initiating methionine	UNP Q83AP3
BBB	-20	HIS	-	expression tag	UNP Q83AP3
BBB	-19	HIS	-	expression tag	UNP Q83AP3

Continued on next page...

CONFIDENTIAL VALIDATION REPORT

Continued from previous page...

Chain	Residue	Modelled	Actual	Comment	Reference
BBB	-18	HIS	-	expression tag	UNP Q83AP3
BBB	-17	HIS	-	expression tag	UNP Q83AP3
BBB	-16	HIS	-	expression tag	UNP Q83AP3
BBB	-15	HIS	-	expression tag	UNP Q83AP3
BBB	-14	SER	-	expression tag	UNP Q83AP3
BBB	-13	SER	-	expression tag	UNP Q83AP3
BBB	-12	GLY	-	expression tag	UNP Q83AP3
BBB	-11	VAL	-	expression tag	UNP Q83AP3
BBB	-10	ASP	-	expression tag	UNP Q83AP3
BBB	-9	LEU	-	expression tag	UNP Q83AP3
BBB	-8	GLY	-	expression tag	UNP Q83AP3
BBB	-7	THR	-	expression tag	UNP Q83AP3
BBB	-6	GLU	-	expression tag	UNP Q83AP3
BBB	-5	ASN	-	expression tag	UNP Q83AP3
BBB	-4	LEU	-	expression tag	UNP Q83AP3
BBB	-3	TYR	-	expression tag	UNP Q83AP3
BBB	-2	PHE	-	expression tag	UNP Q83AP3
BBB	-1	GLN	-	expression tag	UNP Q83AP3
BBB	0	SER	-	expression tag	UNP Q83AP3

- Molecule 2 is THYMIDINE-5'-DIPHOSPHATE (three-letter code: TYD) (formula: $C_{10}H_{16}N_2O_{11}P_2$) (labeled as "Ligand of Interest" by depositor).

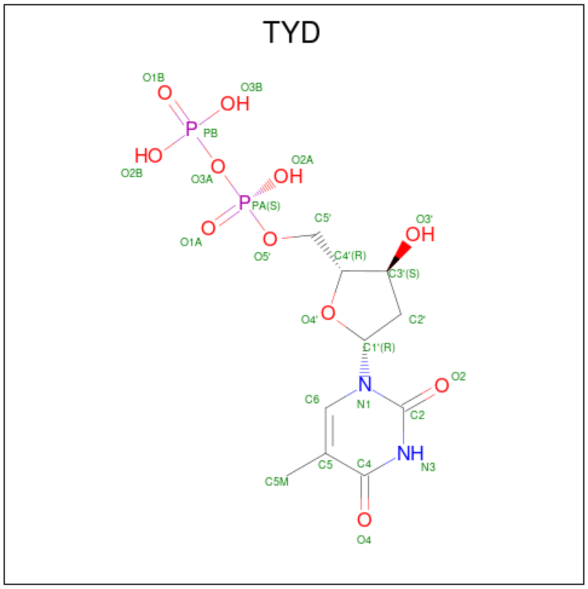

Mol	Chain	Residues	Atoms					ZeroOcc	AltConf
			Total	C	N	O	P		
2	AAA	1	25	10	2	11	2	0	0

Continued on next page...

CONFIDENTIAL VALIDATION REPORT

Continued from previous page...

Mol	Chain	Residues	Atoms					ZeroOcc	AltConf
2	AAA	1	Total 50	C 20	N 4	O 22	P 4	0	1
2	BBB	1	Total 25	C 10	N 2	O 11	P 2	0	0
2	BBB	1	Total 50	C 20	N 4	O 22	P 4	0	1

- Molecule 3 is CITRIC ACID (three-letter code: CIT) (formula: $C_6H_8O_7$).

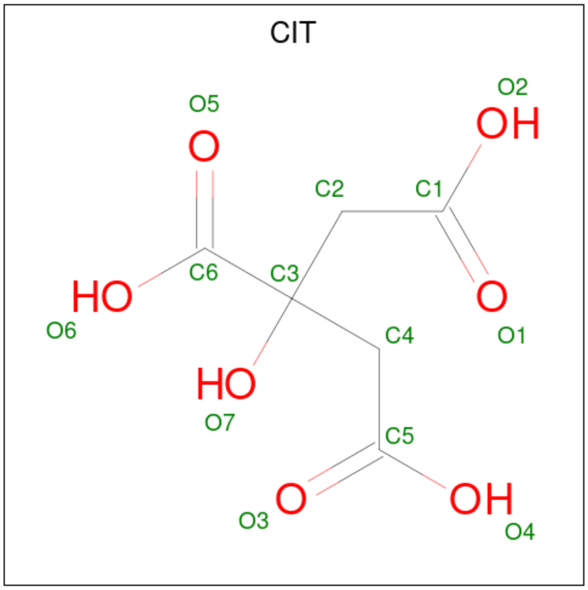

Mol	Chain	Residues	Atoms			ZeroOcc	AltConf
3	AAA	1	Total 13	C 6	O 7	0	0
3	BBB	1	Total 13	C 6	O 7	0	0

- Molecule 4 is 1,2-ETHANEDIOL (three-letter code: EDO) (formula: $C_2H_6O_2$).

CONFIDENTIAL VALIDATION REPORT

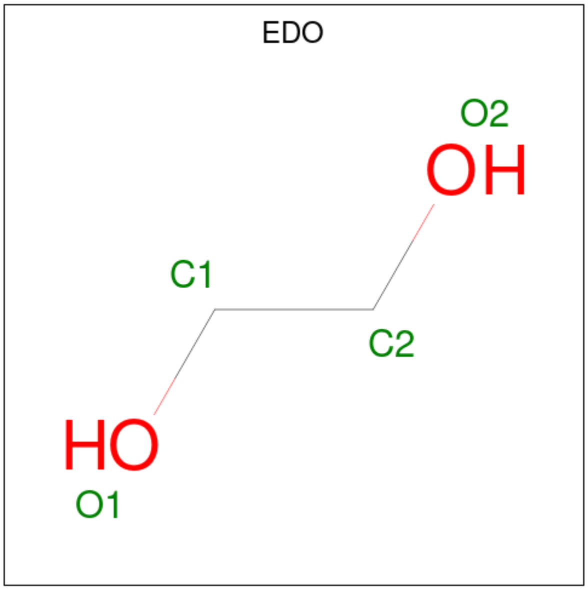

Mol	Chain	Residues	Atoms	ZeroOcc	AltConf
4	AAA	1	Total C O<br>4 2 2	0	0
4	AAA	1	Total C O<br>4 2 2	0	0
4	AAA	1	Total C O<br>4 2 2	0	0
4	BBB	1	Total C O<br>4 2 2	0	0
4	BBB	1	Total C O<br>4 2 2	0	0
4	BBB	1	Total C O<br>4 2 2	0	0
4	BBB	1	Total C O<br>4 2 2	0	0

- Molecule 5 is water.

Mol	Chain	Residues	Atoms	ZeroOcc	AltConf
5	AAA	114	Total O<br>114 114	0	0
5	BBB	124	Total O<br>124 124	0	0

CONFIDENTIAL VALIDATION REPORT

3 Residue-property plots (i)

These plots are drawn for all protein, RNA, DNA and oligosaccharide chains in the entry. The first graphic for a chain summarises the proportions of the various outlier classes displayed in the second graphic. The second graphic shows the sequence view annotated by issues in geometry and electron density. Residues are color-coded according to the number of geometric quality criteria for which they contain at least one outlier: green = 0, yellow = 1, orange = 2 and red = 3 or more. A red dot above a residue indicates a poor fit to the electron density (RSRZ > 2). Stretches of 2 or more consecutive residues without any outlier are shown as a green connector. Residues present in the sample, but not in the model, are shown in grey.

- Molecule 1: dTDP-4-dehydrorhamnose 3,5-epimerase

Chain AAA:

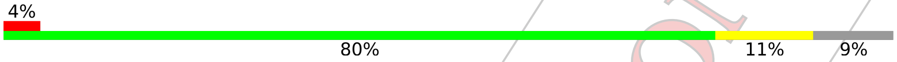

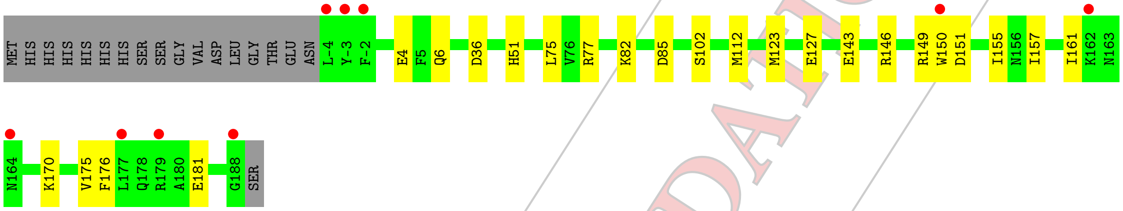

- Molecule 1: dTDP-4-dehydrorhamnose 3,5-epimerase

Chain BBB:

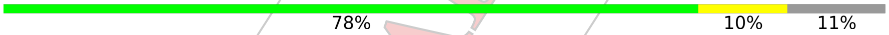

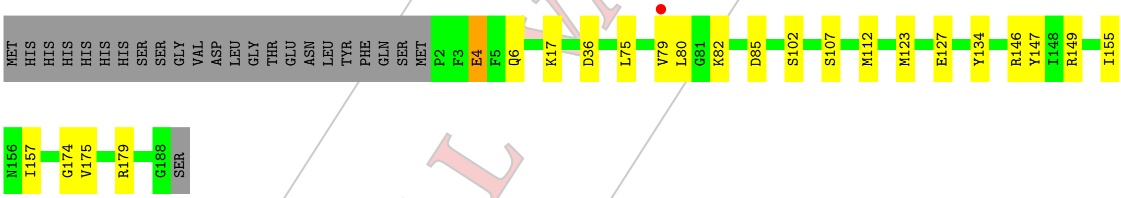

CONFIDENTIAL VALIDATION REPORT

4 Data and refinement statistics (i)

Property	Value	Source
Space group	C 2 2 21	Depositor
Cell constants<br>a, b, c, α, β, γ	78.88Å 82.15Å 163.83Å<br>90.00° 90.00° 90.00°	Depositor
Resolution (Å)	81.92 – 1.87<br>81.92 – 1.87	Depositor<br>EDS
% Data completeness<br>(in resolution range)	84.4 (81.92-1.87)<br>84.4 (81.92-1.87)	Depositor<br>EDS
R_{merge}	(Not available)	Depositor
R_{sym}	(Not available)	Depositor
$< I/\sigma(I) >$ [1]	1.51 (at 1.87Å)	Xtriage
Refinement program	REFMAC 5.8.0267	Depositor
R, R_{free}	0.209 , 0.237<br>0.214 , 0.242	Depositor<br>DCC
R_{free} test set	1856 reflections (4.96%)	wwPDB-VP
Wilson B-factor (Å^2)	40.3	Xtriage
Anisotropy	0.032	Xtriage
Bulk solvent k_{sol}(e/Å^3), B_{sol}(Å^2)	(Not available) , (Not available)	EDS
L-test for twinning[2]	$< \|L\| > = 0.50$, $< L^2 > = 0.34$	Xtriage
Estimated twinning fraction	0.025 for -k,-h,-l	Xtriage
F_o,F_c correlation	0.96	EDS
Total number of atoms	3766	wwPDB-VP
Average B, all atoms (Å^2)	43.0	wwPDB-VP

Xtriage's analysis on translational NCS is as follows: *The largest off-origin peak in the Patterson function is 4.79% of the height of the origin peak. No significant pseudotranslation is detected.*

[1] Intensities estimated from amplitudes.
[2] Theoretical values of $< |L| >$, $< L^2 >$ for acentric reflections are 0.5, 0.333 respectively for untwinned datasets, and 0.375, 0.2 for perfectly twinned datasets.

CONFIDENTIAL VALIDATION REPORT

5 Model quality (i)

5.1 Standard geometry (i)

Bond lengths and bond angles in the following residue types are not validated in this section: CIT, TYD, EDO

The Z score for a bond length (or angle) is the number of standard deviations the observed value is removed from the expected value. A bond length (or angle) with $|Z| > 5$ is considered an outlier worth inspection. RMSZ is the root-mean-square of all Z scores of the bond lengths (or angles).

Mol	Chain	Bond lengths		Bond angles	
		RMSZ	$\#\|Z\|>5$	RMSZ	$\#\|Z\|>5$
1	AAA	0.67	0/1813	0.86	0/2446
1	BBB	0.68	0/1701	0.89	0/2295
All	All	0.68	0/3514	0.88	0/4741

There are no bond length outliers.

There are no bond angle outliers.

There are no chirality outliers.

There are no planarity outliers.

5.2 Too-close contacts (i)

In the following table, the Non-H and H(model) columns list the number of non-hydrogen atoms and hydrogen atoms in the chain respectively. The H(added) column lists the number of hydrogen atoms added and optimized by MolProbity. The Clashes column lists the number of clashes within the asymmetric unit, whereas Symm-Clashes lists symmetry-related clashes.

Mol	Chain	Non-H	H(model)	H(added)	Clashes	Symm-Clashes
1	AAA	1707	0	1760	23	1
1	BBB	1617	0	1654	28	0
2	AAA	75	0	39	2	0
2	BBB	75	0	39	2	0
3	AAA	13	0	5	1	0
3	BBB	13	0	5	2	0
4	AAA	12	0	18	4	0
4	BBB	16	0	24	5	0
5	AAA	114	0	0	3	0
5	BBB	124	0	0	0	0
All	All	3766	0	3544	54	1

CONFIDENTIAL VALIDATION REPORT

The all-atom clashscore is defined as the number of clashes found per 1000 atoms (including hydrogen atoms). The all-atom clashscore for this structure is 8.

All (54) close contacts within the same asymmetric unit are listed below, sorted by their clash magnitude.

Atom-1	Atom-2	Interatomic distance (Å)	Clash overlap (Å)
1:BBB:4[B]:GLU:OE2	1:BBB:6[B]:GLN:NE2	1.67	1.28
1:BBB:75[B]:LEU:HD11	1:BBB:112[B]:MET:SD	1.90	1.11
1:AAA:4[B]:GLU:OE1	1:AAA:6[B]:GLN:OE1	1.74	1.06
1:BBB:4[B]:GLU:CD	1:BBB:6[B]:GLN:HE21	1.66	0.99
1:AAA:146:ARG:HD2	4:AAA:205:EDO:H22	1.52	0.91
1:BBB:75[B]:LEU:CD1	1:BBB:112[B]:MET:SD	2.61	0.88
1:AAA:4[A]:GLU:HG2	5:AAA:408:HOH:O	1.81	0.80
1:BBB:147:TYR:HB2	4:BBB:204:EDO:H12	1.69	0.74
1:BBB:75[B]:LEU:CG	1:BBB:112[B]:MET:SD	2.78	0.71
1:AAA:75[B]:LEU:HD11	1:AAA:112[B]:MET:SD	2.31	0.71
1:AAA:75[B]:LEU:CG	1:AAA:112[B]:MET:SD	2.80	0.69
1:AAA:75[B]:LEU:HG	1:AAA:112[B]:MET:SD	2.33	0.69
1:AAA:75[B]:LEU:HD21	1:AAA:112[B]:MET:SD	2.37	0.65
1:BBB:4[A]:GLU:HG3	1:BBB:17:LYS:HB2	1.81	0.63
2:AAA:202[A]:TYD:H5'2	2:AAA:202[A]:TYD:H6	1.81	0.63
1:AAA:146:ARG:CD	4:AAA:205:EDO:H22	2.28	0.63
2:BBB:202[A]:TYD:H6	2:BBB:202[A]:TYD:H5'1	1.81	0.62
1:AAA:77[A]:ARG:HD3	5:AAA:337:HOH:O	1.99	0.62
2:AAA:201:TYD:O3B	3:AAA:203:CIT:O1	2.18	0.60
1:BBB:75[B]:LEU:HG	1:BBB:112[B]:MET:SD	2.40	0.60
1:BBB:75[B]:LEU:CD1	1:BBB:112[B]:MET:CG	2.80	0.59
1:BBB:75[B]:LEU:HD11	1:BBB:112[B]:MET:CG	2.32	0.59
1:AAA:75[B]:LEU:CD1	1:AAA:112[B]:MET:SD	2.92	0.57
2:BBB:201:TYD:O1B	3:BBB:203:CIT:O2	2.23	0.56
1:BBB:75[B]:LEU:CD1	1:BBB:112[B]:MET:HG2	2.35	0.56
1:BBB:75[B]:LEU:HD21	1:BBB:112[B]:MET:SD	2.49	0.53
1:AAA:155:ILE:HG22	1:AAA:157:ILE:HG13	1.91	0.52
1:AAA:75[B]:LEU:CD2	1:AAA:112[B]:MET:SD	2.97	0.52
1:BBB:146:ARG:HB3	4:BBB:206:EDO:H22	1.94	0.50
1:AAA:149:ARG:HD3	1:AAA:176:PHE:CE2	2.46	0.50
1:BBB:155:ILE:HG22	1:BBB:157:ILE:HG13	1.95	0.49
1:AAA:175:VAL:HG21	4:AAA:205:EDO:H12	1.94	0.49
1:AAA:146:ARG:HD2	4:AAA:205:EDO:C2	2.35	0.48
1:BBB:75[B]:LEU:HD12	1:BBB:112[B]:MET:HG2	1.96	0.48
1:AAA:149:ARG:HD3	1:AAA:176:PHE:CZ	2.48	0.48
1:BBB:134:TYR:OH	3:BBB:203:CIT:H21	2.16	0.46
1:BBB:146:ARG:HD2	4:BBB:206:EDO:H11	1.99	0.45

Continued on next page...

CONFIDENTIAL VALIDATION REPORT

Continued from previous page...

Atom-1	Atom-2	Interatomic distance (Å)	Clash overlap (Å)
1:AAA:181:GLU:HA	5:AAA:301:HOH:O	2.16	0.45
1:AAA:82:LYS:HE2	1:AAA:127:GLU:OE2	2.17	0.45
1:BBB:82:LYS:HE3	1:BBB:127:GLU:OE2	2.17	0.44
1:BBB:4[B]:GLU:CD	1:BBB:6[B]:GLN:NE2	2.45	0.44
1:BBB:149[A]:ARG:NE	1:BBB:174:GLY:O	2.45	0.44
1:BBB:147:TYR:CB	4:BBB:204:EDO:H12	2.43	0.43
1:BBB:75[B]:LEU:HG	1:BBB:112[B]:MET:CE	2.49	0.43
1:BBB:75[B]:LEU:CD2	1:BBB:112[B]:MET:SD	3.07	0.43
1:AAA:150[B]:TRP:CE3	1:AAA:151:ASP:HB3	2.54	0.42
1:BBB:175:VAL:HG13	4:BBB:204:EDO:O2	2.19	0.42
1:AAA:85:ASP:O	1:AAA:102:SER:HA	2.20	0.42
1:AAA:51:HIS:CE1	1:BBB:79[A]:VAL:HG23	2.55	0.41
1:AAA:170[A]:LYS:HB2	1:AAA:170[A]:LYS:HE3	1.77	0.41
1:BBB:85:ASP:O	1:BBB:102:SER:HA	2.21	0.41
1:BBB:75[B]:LEU:HD11	1:BBB:112[B]:MET:HG2	1.97	0.41
1:BBB:80[B]:LEU:HA	1:BBB:107:SER:HB3	2.01	0.41

All (1) symmetry-related close contacts are listed below. The label for Atom-2 includes the symmetry operator and encoded unit-cell translations to be applied.

Atom-1	Atom-2	Interatomic distance (Å)	Clash overlap (Å)
1:AAA:151:ASP:OD2	1:AAA:151:ASP:OD2[4_545]	1.97	0.23

5.3 Torsion angles (i)

5.3.1 Protein backbone (i)

In the following table, the Percentiles column shows the percent Ramachandran outliers of the chain as a percentile score with respect to all X-ray entries followed by that with respect to entries of similar resolution.

The Analysed column shows the number of residues for which the backbone conformation was analysed, and the total number of residues.

Mol	Chain	Analysed	Favoured	Allowed	Outliers	Percentiles	
1	AAA	211/211 (100%)	206 (98%)	4 (2%)	1 (0%)	29	17
1	BBB	199/211 (94%)	195 (98%)	4 (2%)	0	100	100
All	All	410/422 (97%)	401 (98%)	8 (2%)	1 (0%)	41	37

CONFIDENTIAL VALIDATION REPORT

All (1) Ramachandran outliers are listed below:

Mol	Chain	Res	Type
1	AAA	161	ILE

5.3.2 Protein sidechains (i)

In the following table, the Percentiles column shows the percent sidechain outliers of the chain as a percentile score with respect to all X-ray entries followed by that with respect to entries of similar resolution.

The Analysed column shows the number of residues for which the sidechain conformation was analysed, and the total number of residues.

Mol	Chain	Analysed	Rotameric	Outliers	Percentiles	
1	AAA	193/189 (102%)	191 (99%)	2 (1%)	76	73
1	BBB	181/189 (96%)	175 (97%)	6 (3%)	38	26
All	All	374/378 (99%)	366 (98%)	8 (2%)	59	45

All (8) residues with a non-rotameric sidechain are listed below:

Mol	Chain	Res	Type
1	AAA	36	ASP
1	AAA	123	MET
1	BBB	4[A]	GLU
1	BBB	4[B]	GLU
1	BBB	36	ASP
1	BBB	123	MET
1	BBB	179[A]	ARG
1	BBB	179[B]	ARG

Sometimes sidechains can be flipped to improve hydrogen bonding and reduce clashes. There are no such sidechains identified.

5.3.3 RNA (i)

There are no RNA molecules in this entry.

5.4 Non-standard residues in protein, DNA, RNA chains (i)

There are no non-standard protein/DNA/RNA residues in this entry.

CONFIDENTIAL VALIDATION REPORT

5.5 Carbohydrates (i)

There are no monosaccharides in this entry.

5.6 Ligand geometry (i)

15 ligands are modelled in this entry.

In the following table, the Counts columns list the number of bonds (or angles) for which Mogul statistics could be retrieved, the number of bonds (or angles) that are observed in the model and the number of bonds (or angles) that are defined in the Chemical Component Dictionary. The Link column lists molecule types, if any, to which the group is linked. The Z score for a bond length (or angle) is the number of standard deviations the observed value is removed from the expected value. A bond length (or angle) with $|Z| > 2$ is considered an outlier worth inspection. RMSZ is the root-mean-square of all Z scores of the bond lengths (or angles).

Mol	Type	Chain	Res	Link	Bond lengths			Bond angles		
					Counts	RMSZ	$\#\|Z\| > 2$	Counts	RMSZ	$\#\|Z\| > 2$
4	EDO	BBB	206	-	3,3,3	0.24	0	2,2,2	0.21	0
4	EDO	BBB	204	-	3,3,3	0.11	0	2,2,2	0.54	0
2	TYD	BBB	202[A]	-	23,26,26	1.99	2 (8%)	33,40,40	1.58	3 (9%)
2	TYD	BBB	202[B]	2	23,26,26	1.99	4 (17%)	33,40,40	1.65	4 (12%)
3	CIT	BBB	203	-	3,12,12	0.55	0	3,17,17	0.95	0
2	TYD	AAA	201	-	23,26,26	1.94	2 (8%)	33,40,40	1.71	5 (15%)
3	CIT	AAA	203	-	3,12,12	0.44	0	3,17,17	1.59	1 (33%)
4	EDO	AAA	204	-	3,3,3	0.16	0	2,2,2	0.55	0
4	EDO	BBB	205	-	3,3,3	0.16	0	2,2,2	0.47	0
2	TYD	BBB	201	-	23,26,26	1.95	3 (13%)	33,40,40	1.64	3 (9%)
4	EDO	AAA	205	-	3,3,3	0.16	0	2,2,2	0.58	0
4	EDO	AAA	206	-	3,3,3	0.34	0	2,2,2	0.57	0
2	TYD	AAA	202[A]	-	23,26,26	1.93	4 (17%)	33,40,40	1.58	4 (12%)
2	TYD	AAA	202[B]	2	23,26,26	1.93	4 (17%)	33,40,40	1.63	3 (9%)
4	EDO	BBB	207	-	3,3,3	0.09	0	2,2,2	0.11	0

In the following table, the Chirals column lists the number of chiral outliers, the number of chiral centers analysed, the number of these observed in the model and the number defined in the Chemical Component Dictionary. Similar counts are reported in the Torsion and Rings columns. '-' means no outliers of that kind were identified.

Mol	Type	Chain	Res	Link	Chirals	Torsions	Rings
4	EDO	BBB	206	-	-	0/1/1/1	-
4	EDO	BBB	204	-	-	0/1/1/1	-
2	TYD	BBB	202[A]	-	-	4/16/28/28	0/2/2/2

Continued on next page...

CONFIDENTIAL VALIDATION REPORT

Continued from previous page...

Mol	Type	Chain	Res	Link	Chirals	Torsions	Rings
2	TYD	BBB	202[B]	2	-	7/16/28/28	0/2/2/2
3	CIT	BBB	203	-	-	0/6/16/16	-
2	TYD	AAA	201	-	-	1/16/28/28	0/2/2/2
3	CIT	AAA	203	-	-	0/6/16/16	-
4	EDO	AAA	204	-	-	1/1/1/1	-
4	EDO	BBB	205	-	-	1/1/1/1	-
2	TYD	BBB	201	-	-	1/16/28/28	0/2/2/2
4	EDO	AAA	205	-	-	0/1/1/1	-
4	EDO	AAA	206	-	-	1/1/1/1	-
2	TYD	AAA	202[A]	-	-	5/16/28/28	0/2/2/2
2	TYD	AAA	202[B]	2	-	10/16/28/28	0/2/2/2
4	EDO	BBB	207	-	-	0/1/1/1	-

All (19) bond length outliers are listed below:

Mol	Chain	Res	Type	Atoms	Z	Observed(Å)	Ideal(Å)
2	BBB	202[A]	TYD	C6-N1	-7.02	1.37	1.46
2	BBB	202[B]	TYD	C6-N1	-6.60	1.38	1.46
2	AAA	201	TYD	C6-N1	-6.58	1.38	1.46
2	BBB	201	TYD	C6-N1	-6.55	1.38	1.46
2	AAA	202[B]	TYD	C6-N1	-6.49	1.38	1.46
2	AAA	202[A]	TYD	C6-N1	-6.40	1.38	1.46
2	BBB	202[B]	TYD	C6-C5	-5.54	1.34	1.51
2	BBB	202[A]	TYD	C6-C5	-5.45	1.34	1.51
2	AAA	202[A]	TYD	C6-C5	-5.40	1.35	1.51
2	AAA	202[B]	TYD	C6-C5	-5.39	1.35	1.51
2	AAA	201	TYD	C6-C5	-5.35	1.35	1.51
2	BBB	201	TYD	C6-C5	-5.33	1.35	1.51
2	BBB	201	TYD	C2-N1	2.75	1.39	1.35
2	AAA	202[A]	TYD	C1'-N1	2.41	1.48	1.45
2	BBB	202[B]	TYD	C1'-N1	2.40	1.48	1.45
2	BBB	202[B]	TYD	C2-N1	2.32	1.38	1.35
2	AAA	202[B]	TYD	C1'-N1	2.21	1.48	1.45
2	AAA	202[A]	TYD	C2-N1	2.14	1.38	1.35
2	AAA	202[B]	TYD	C2-N1	2.10	1.38	1.35

All (23) bond angle outliers are listed below:

Mol	Chain	Res	Type	Atoms	Z	Observed(°)	Ideal(°)
2	AAA	201	TYD	C5-C6-N1	6.42	123.76	111.11
2	BBB	202[B]	TYD	C5-C6-N1	6.33	123.59	111.11

Continued on next page...

CONFIDENTIAL VALIDATION REPORT

Continued from previous page...

Mol	Chain	Res	Type	Atoms	Z	Observed(°)	Ideal(°)
2	AAA	202[B]	TYD	C5-C6-N1	6.23	123.39	111.11
2	BBB	201	TYD	C5-C6-N1	6.19	123.31	111.11
2	AAA	202[A]	TYD	C5-C6-N1	6.16	123.25	111.11
2	BBB	202[A]	TYD	C5-C6-N1	6.14	123.20	111.11
2	BBB	201	TYD	C5M-C5-C6	5.30	123.65	112.34
2	BBB	202[B]	TYD	C5M-C5-C6	4.97	122.96	112.34
2	AAA	202[A]	TYD	C5M-C5-C6	4.96	122.92	112.34
2	AAA	202[B]	TYD	C5M-C5-C6	4.92	122.85	112.34
2	AAA	201	TYD	C5M-C5-C6	4.82	122.64	112.34
2	BBB	202[A]	TYD	C5M-C5-C6	4.40	121.75	112.34
3	AAA	203	CIT	C3-C2-C1	2.68	119.27	114.98
2	AAA	201	TYD	C6-C5-C4	2.51	118.74	111.53
2	BBB	202[A]	TYD	C6-C5-C4	2.38	118.37	111.53
2	AAA	202[A]	TYD	C6-C5-C4	2.38	118.35	111.53
2	BBB	202[B]	TYD	C6-C5-C4	2.38	118.35	111.53
2	BBB	201	TYD	C6-C5-C4	2.37	118.33	111.53
2	AAA	202[B]	TYD	C6-C5-C4	2.33	118.22	111.53
2	BBB	202[B]	TYD	N3-C2-N1	-2.14	114.39	116.65
2	AAA	201	TYD	O2A-PA-O1A	2.12	122.72	112.24
2	AAA	201	TYD	O3B-PB-O2B	2.03	115.39	107.64
2	AAA	202[A]	TYD	N3-C2-N1	-2.00	114.53	116.65

There are no chirality outliers.

All (31) torsion outliers are listed below:

Mol	Chain	Res	Type	Atoms
2	AAA	201	TYD	PA-O3A-PB-O3B
2	AAA	202[A]	TYD	C5'-O5'-PA-O1A
2	AAA	202[A]	TYD	C5'-O5'-PA-O3A
2	AAA	202[A]	TYD	C3'-C4'-C5'-O5'
2	AAA	202[B]	TYD	C5'-O5'-PA-O1A
2	AAA	202[B]	TYD	C5'-O5'-PA-O3A
2	AAA	202[B]	TYD	C4'-C5'-O5'-PA
2	AAA	202[B]	TYD	O4'-C1'-N1-C6
2	BBB	202[A]	TYD	C5'-O5'-PA-O1A
2	BBB	202[A]	TYD	C5'-O5'-PA-O2A
2	BBB	202[B]	TYD	C5'-O5'-PA-O3A
2	BBB	202[B]	TYD	C4'-C5'-O5'-PA
2	BBB	202[B]	TYD	O4'-C1'-N1-C6
2	BBB	202[B]	TYD	O4'-C1'-N1-C2
2	AAA	202[B]	TYD	O4'-C4'-C5'-O5'
2	BBB	202[B]	TYD	C2'-C1'-N1-C6

Continued on next page...

CONFIDENTIAL VALIDATION REPORT

Continued from previous page...

Mol	Chain	Res	Type	Atoms
2	AAA	202[B]	TYD	C2'-C1'-N1-C6
2	AAA	202[B]	TYD	C2'-C1'-N1-C2
2	AAA	202[A]	TYD	O4'-C4'-C5'-O5'
2	AAA	202[B]	TYD	O4'-C1'-N1-C2
4	BBB	205	EDO	O1-C1-C2-O2
2	BBB	202[B]	TYD	C2'-C1'-N1-C2
2	AAA	202[A]	TYD	C4'-C5'-O5'-PA
4	AAA	206	EDO	O1-C1-C2-O2
2	BBB	202[A]	TYD	C5'-O5'-PA-O3A
2	AAA	202[B]	TYD	C5'-O5'-PA-O2A
2	BBB	202[B]	TYD	C5'-O5'-PA-O2A
2	AAA	202[B]	TYD	C3'-C4'-C5'-O5'
2	BBB	201	TYD	PA-O3A-PB-O1B
2	BBB	202[A]	TYD	O4'-C4'-C5'-O5'
4	AAA	204	EDO	O1-C1-C2-O2

There are no ring outliers.

9 monomers are involved in 14 short contacts:

Mol	Chain	Res	Type	Clashes	Symm-Clashes
4	BBB	206	EDO	2	0
4	BBB	204	EDO	3	0
2	BBB	202[A]	TYD	1	0
3	BBB	203	CIT	2	0
2	AAA	201	TYD	1	0
3	AAA	203	CIT	1	0
2	BBB	201	TYD	1	0
4	AAA	205	EDO	4	0
2	AAA	202[A]	TYD	1	0

The following is a two-dimensional graphical depiction of Mogul quality analysis of bond lengths, bond angles, torsion angles, and ring geometry for all instances of the Ligand of Interest. In addition, ligands with molecular weight > 250 and outliers as shown on the validation Tables will also be included. For torsion angles, if less then 5% of the Mogul distribution of torsion angles is within 10 degrees of the torsion angle in question, then that torsion angle is considered an outlier. Any bond that is central to one or more torsion angles identified as an outlier by Mogul will be highlighted in the graph. For rings, the root-mean-square deviation (RMSD) between the ring in question and similar rings identified by Mogul is calculated over all ring torsion angles. If the average RMSD is greater than 60 degrees and the minimal RMSD between the ring in question and any Mogul-identified rings is also greater than 60 degrees, then that ring is considered an outlier. The outliers are highlighted in purple. The color gray indicates Mogul did not find sufficient equivalents in the CSD to analyse the geometry.

CONFIDENTIAL VALIDATION REPORT

Ligand TYD BBB 202 (A)

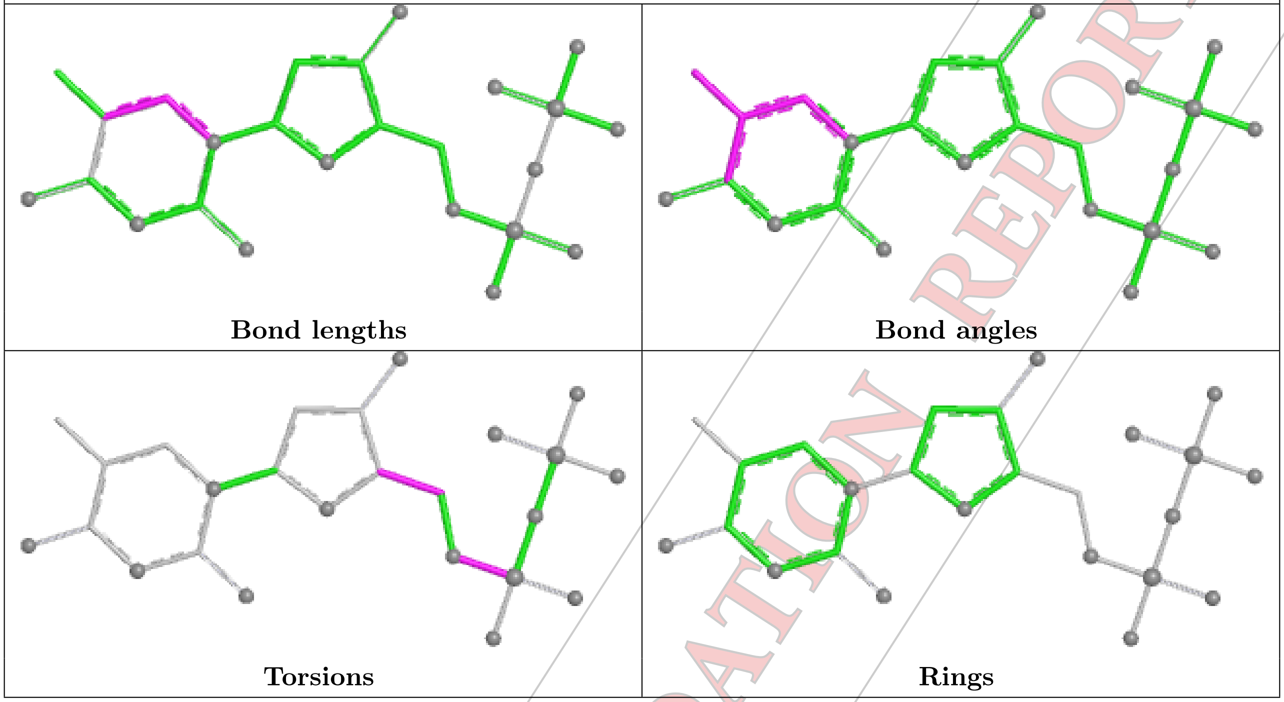

Ligand TYD BBB 202 (B)

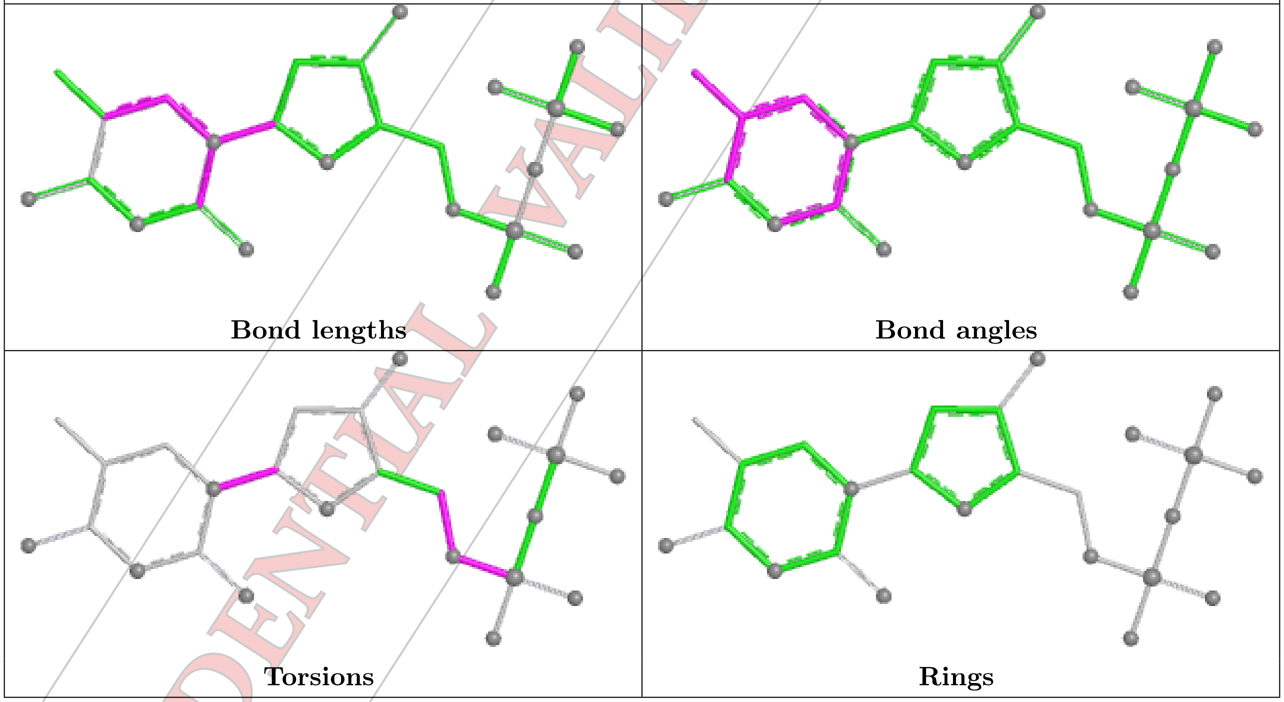

CONFIDENTIAL VALIDATION REPORT

Ligand TYD AAA 201

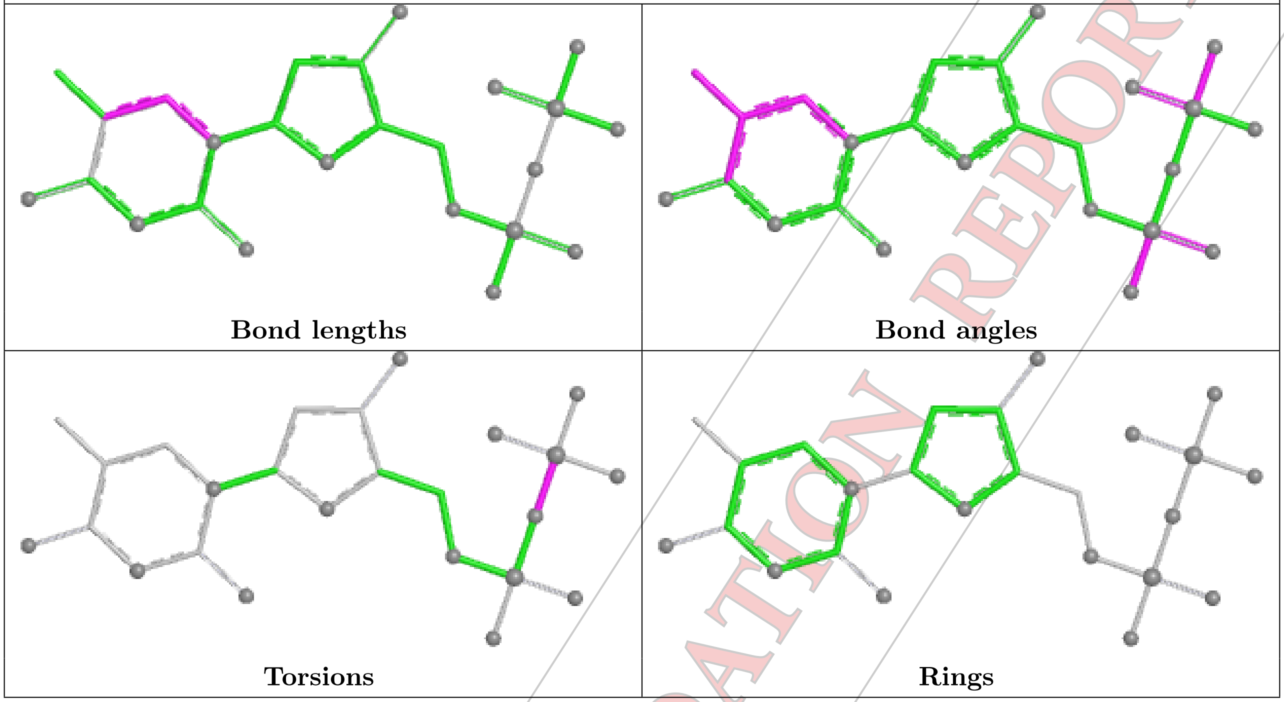

Ligand TYD BBB 201

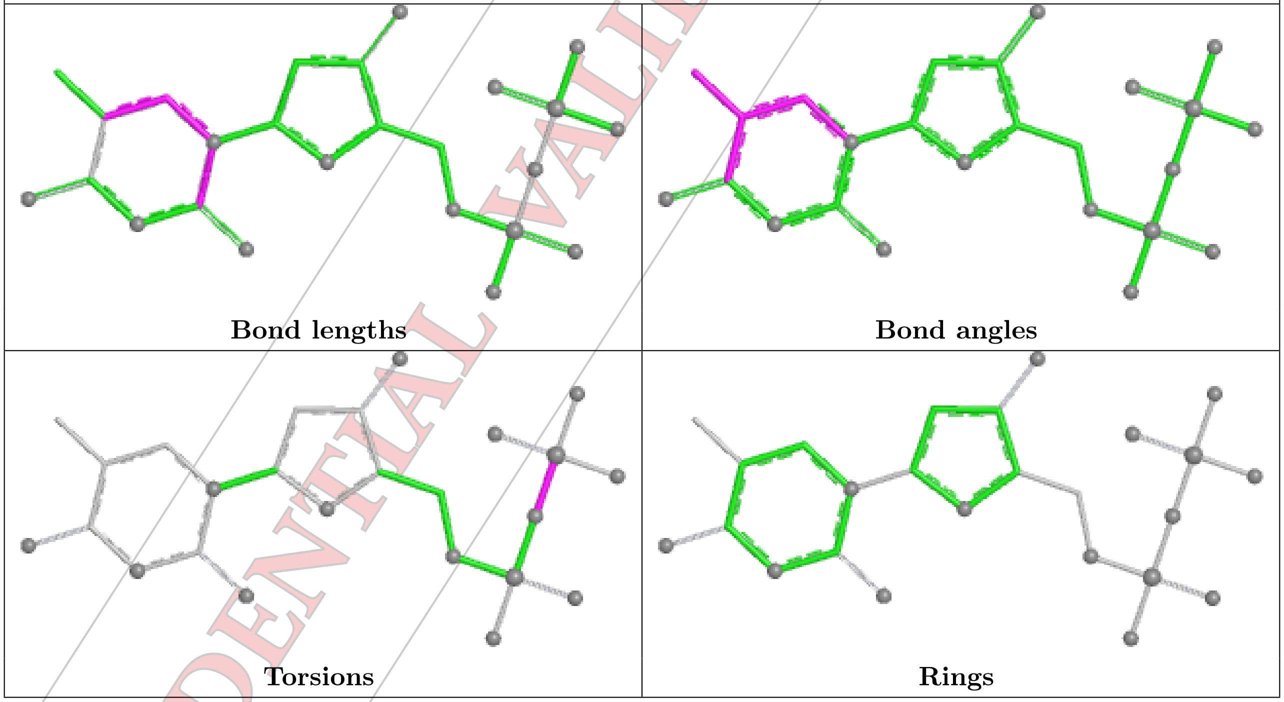

CONFIDENTIAL VALIDATION REPORT

Ligand TYD AAA 202 (A)

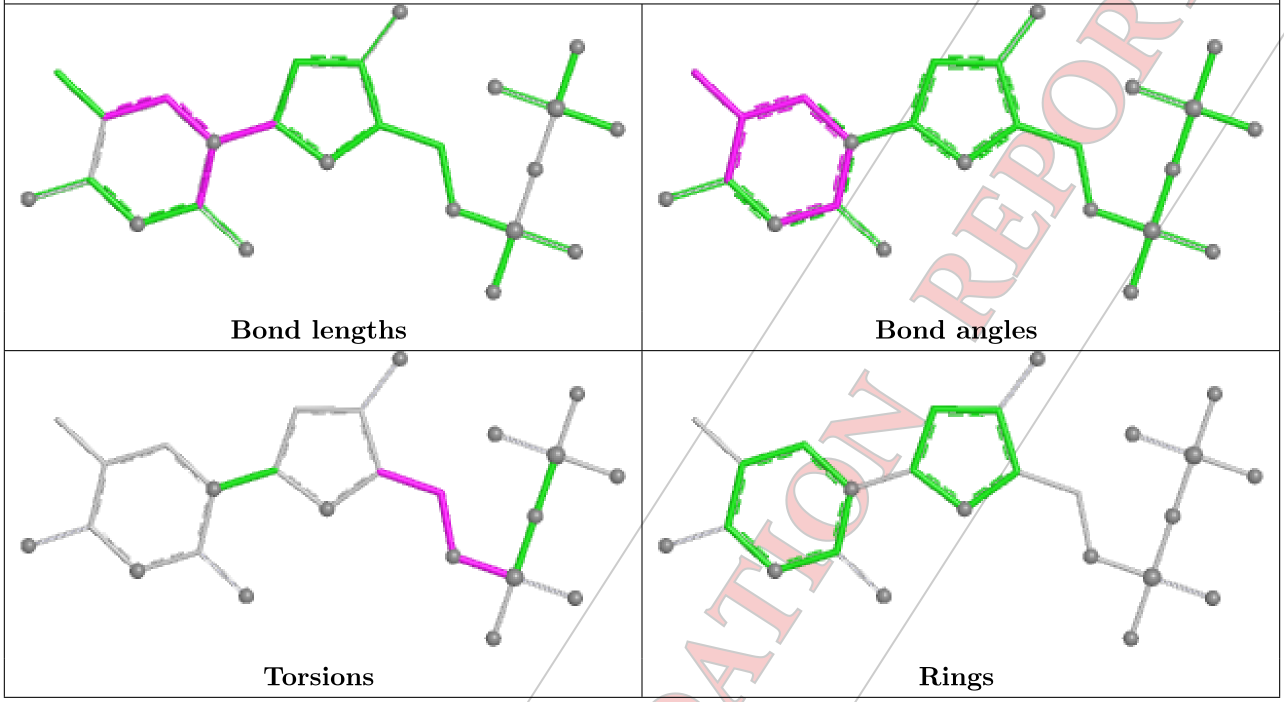

Ligand TYD AAA 202 (B)

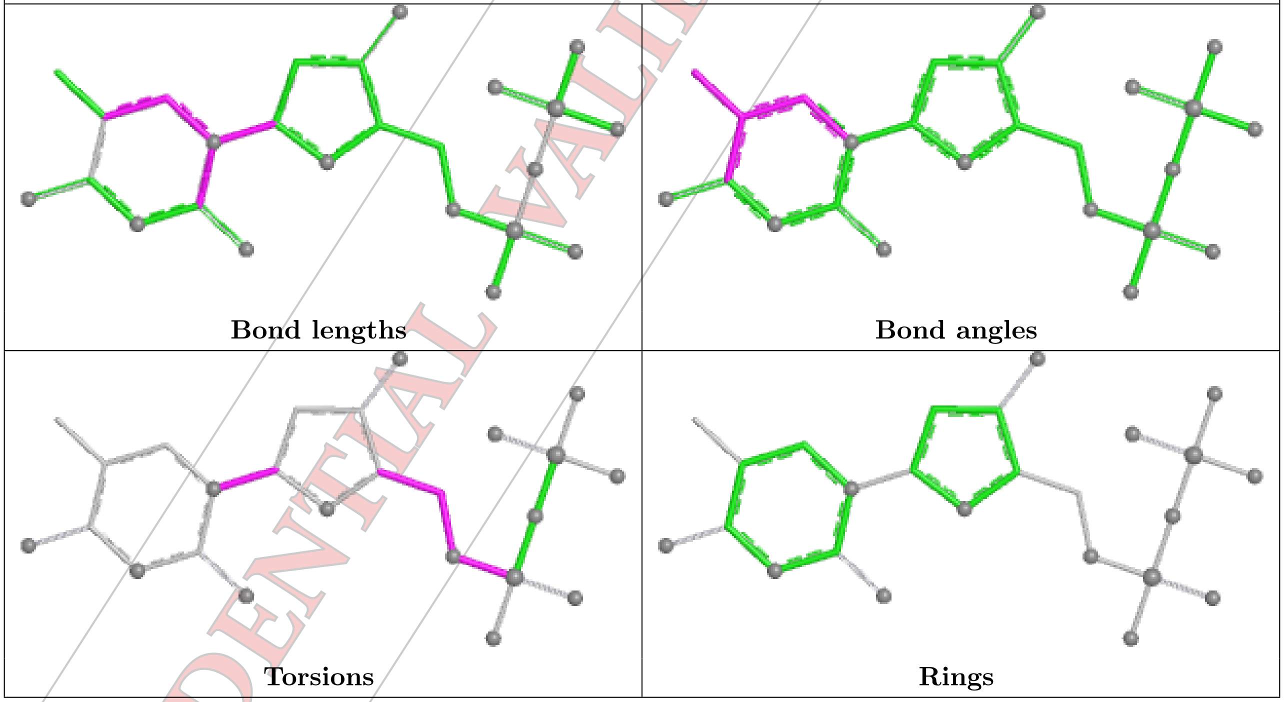

5.7 Other polymers (i)

There are no such residues in this entry.

CONFIDENTIAL VALIDATION REPORT

5.8 Polymer linkage issues (i)

There are no chain breaks in this entry.

CONFIDENTIAL VALIDATION REPORT

6 Fit of model and data (i)

6.1 Protein, DNA and RNA chains (i)

In the following table, the column labelled '#RSRZ> 2' contains the number (and percentage) of RSRZ outliers, followed by percent RSRZ outliers for the chain as percentile scores relative to all X-ray entries and entries of similar resolution. The OWAB column contains the minimum, median, 95th percentile and maximum values of the occupancy-weighted average B-factor per residue. The column labelled 'Q< 0.9' lists the number of (and percentage) of residues with an average occupancy less than 0.9.

Mol	Chain	Analysed	<RSRZ>	#RSRZ>2			OWAB(Å^2)	Q<0.9
1	AAA	193/211 (91%)	0.38	9 (4%)	31	33	26, 39, 62, 102	0
1	BBB	187/211 (88%)	0.22	1 (0%)	91	91	25, 37, 58, 96	0
All	All	380/422 (90%)	0.30	10 (2%)	56	57	25, 38, 61, 102	0

All (10) RSRZ outliers are listed below:

Mol	Chain	Res	Type	RSRZ
1	AAA	-3	TYR	7.4
1	AAA	188	GLY	3.1
1	AAA	-2	PHE	2.8
1	AAA	-4	LEU	2.7
1	AAA	179[A]	ARG	2.7
1	AAA	162	LYS	2.6
1	AAA	150[A]	TRP	2.5
1	AAA	177	LEU	2.2
1	BBB	79[A]	VAL	2.1
1	AAA	164[A]	ASN	2.1

6.2 Non-standard residues in protein, DNA, RNA chains (i)

There are no non-standard protein/DNA/RNA residues in this entry.

6.3 Carbohydrates (i)

There are no monosaccharides in this entry.

CONFIDENTIAL VALIDATION REPORT

6.4 Ligands ⓘ

In the following table, the Atoms column lists the number of modelled atoms in the group and the number defined in the chemical component dictionary. The B-factors column lists the minimum, median, 95th percentile and maximum values of B factors of atoms in the group. The column labelled 'Q< 0.9' lists the number of atoms with occupancy less than 0.9.

Mol	Type	Chain	Res	Atoms	RSCC	RSR	B-factors($Å^2$)	Q<0.9
2	TYD	AAA	202[A]	25/25	0.60	0.38	41,81,88,92	25
2	TYD	AAA	202[B]	25/25	0.60	0.38	73,123,156,165	25
2	TYD	BBB	202[A]	25/25	0.70	0.29	30,65,76,81	25
2	TYD	BBB	202[B]	25/25	0.70	0.29	65,130,165,172	25
4	EDO	AAA	204	4/4	0.71	0.38	56,62,66,69	0
4	EDO	AAA	206	4/4	0.81	0.15	52,55,57,58	0
4	EDO	BBB	205	4/4	0.86	0.15	55,59,62,62	0
4	EDO	BBB	207	4/4	0.86	0.24	77,81,85,88	0
4	EDO	AAA	205	4/4	0.91	0.13	45,50,53,57	0
4	EDO	BBB	204	4/4	0.91	0.27	57,61,65,68	0
4	EDO	BBB	206	4/4	0.92	0.44	44,49,53,55	0
3	CIT	AAA	203	13/13	0.92	0.20	33,47,57,60	0
2	TYD	AAA	201	25/25	0.93	0.10	35,41,46,48	0
3	CIT	BBB	203	13/13	0.96	0.16	30,44,53,54	0
2	TYD	BBB	201	25/25	0.96	0.10	32,38,42,44	0

The following is a graphical depiction of the model fit to experimental electron density of all instances of the Ligand of Interest. In addition, ligands with molecular weight > 250 and outliers as shown on the geometry validation Tables will also be included. Each fit is shown from different orientation to approximate a three-dimensional view.

CONFIDENTIAL VALIDATION REPORT

Electron density around TYD AAA 202 (A):

2mF$_o$-DF$_c$ (at 0.7 rmsd) in gray
mF$_o$-DF$_c$ (at 3 rmsd) in purple (negative)
and green (positive)

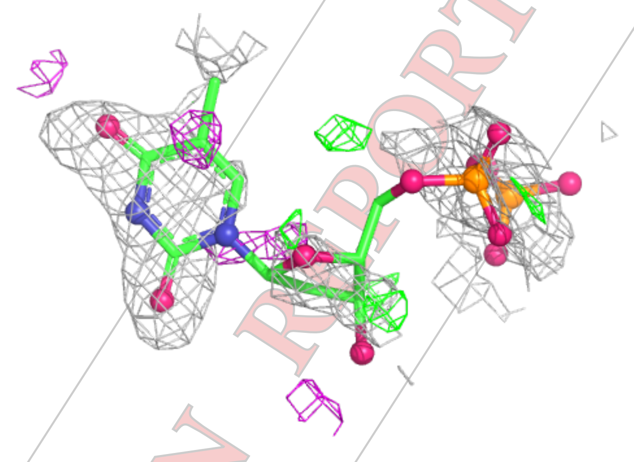

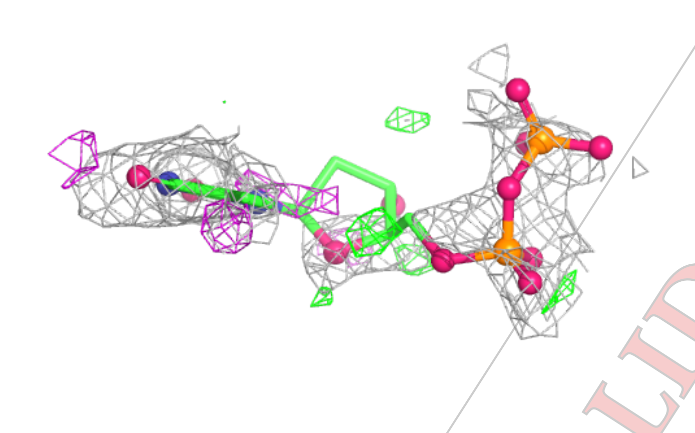

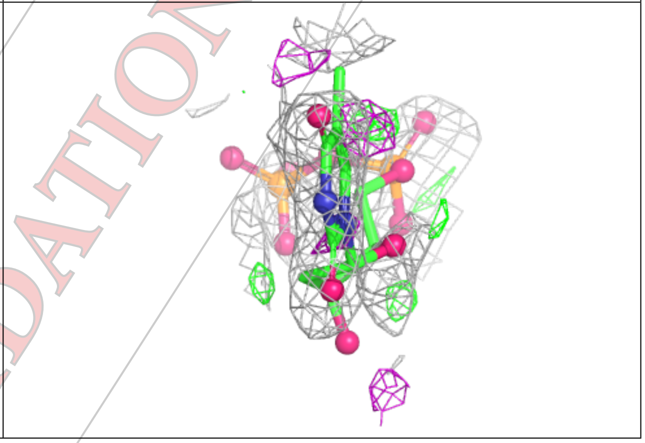

Electron density around TYD AAA 202 (B):

2mF$_o$-DF$_c$ (at 0.7 rmsd) in gray
mF$_o$-DF$_c$ (at 3 rmsd) in purple (negative)
and green (positive)

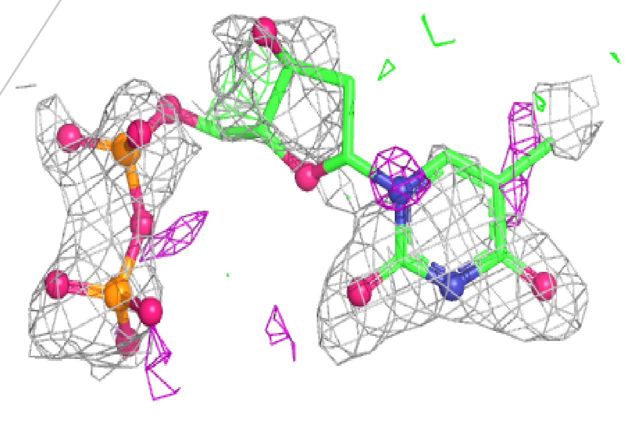

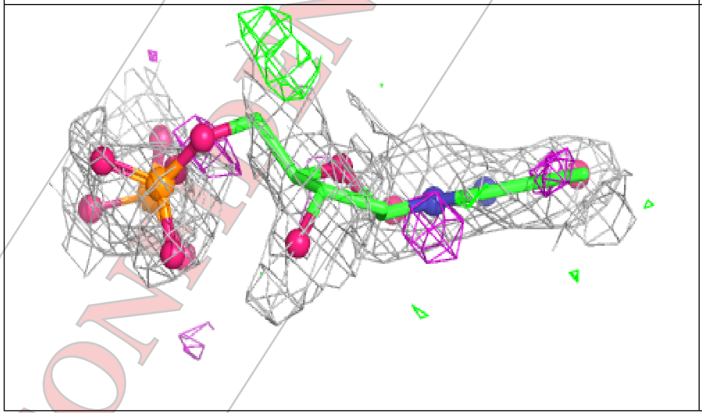

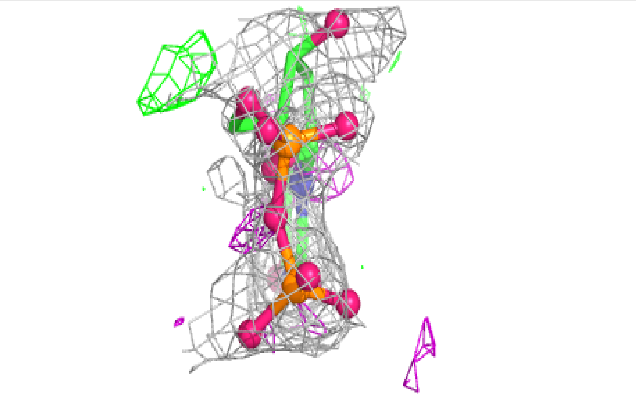

CONFIDENTIAL VALIDATION REPORT

Electron density around TYD BBB 202 (A):

2mF$_o$-DF$_c$ (at 0.7 rmsd) in gray
mF$_o$-DF$_c$ (at 3 rmsd) in purple (negative)
and green (positive)

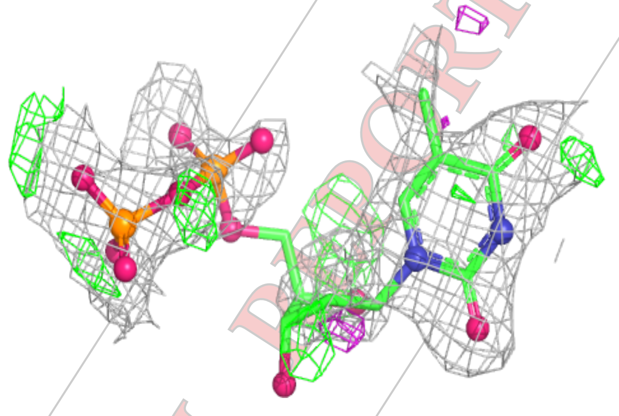

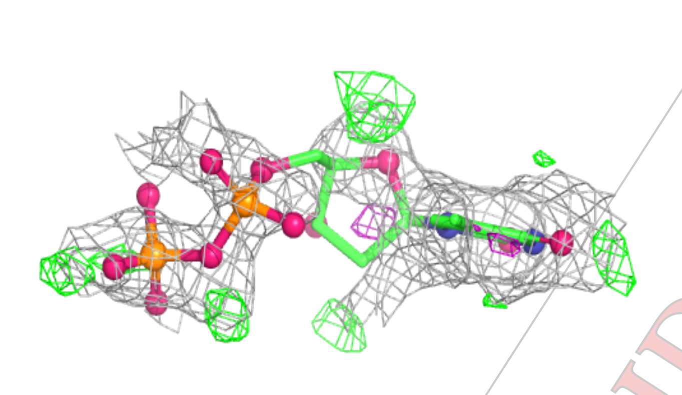

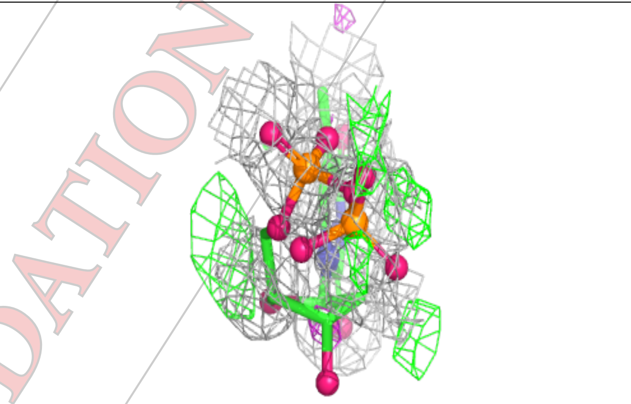

Electron density around TYD BBB 202 (B):

2mF$_o$-DF$_c$ (at 0.7 rmsd) in gray
mF$_o$-DF$_c$ (at 3 rmsd) in purple (negative)
and green (positive)

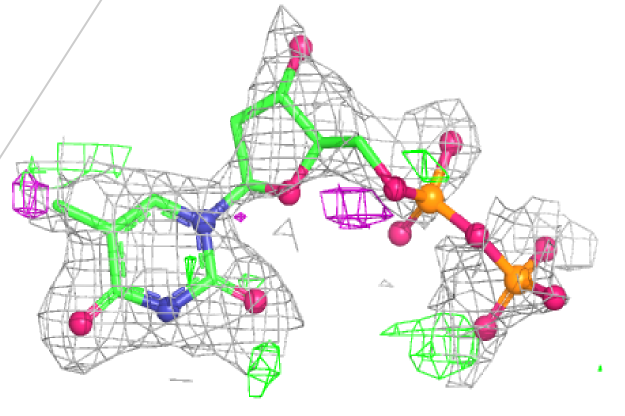

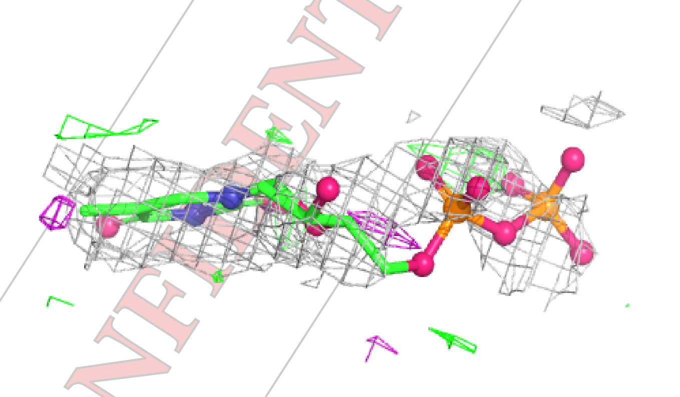

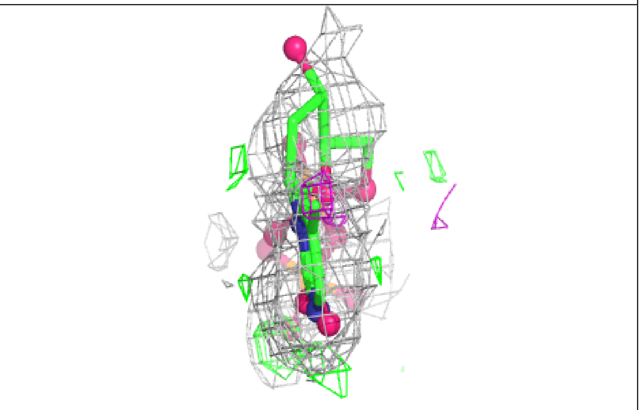

CONFIDENTIAL VALIDATION REPORT

Electron density around TYD AAA 201:

$2mF_o$-DF_c (at 0.7 rmsd) in gray
mF_o-DF_c (at 3 rmsd) in purple (negative)
and green (positive)

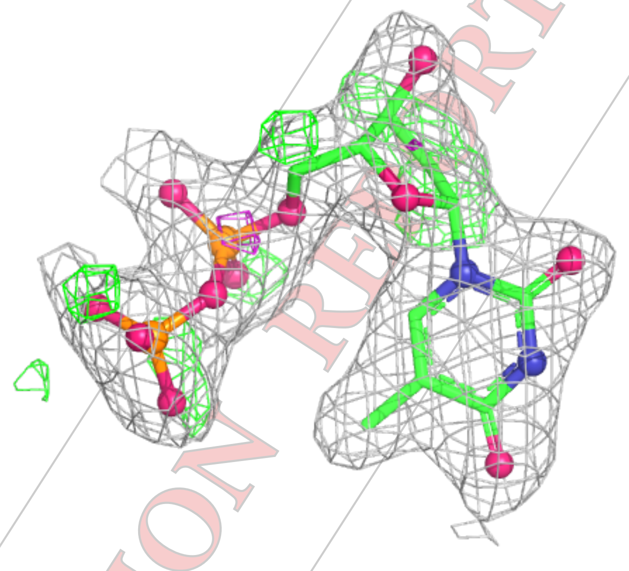

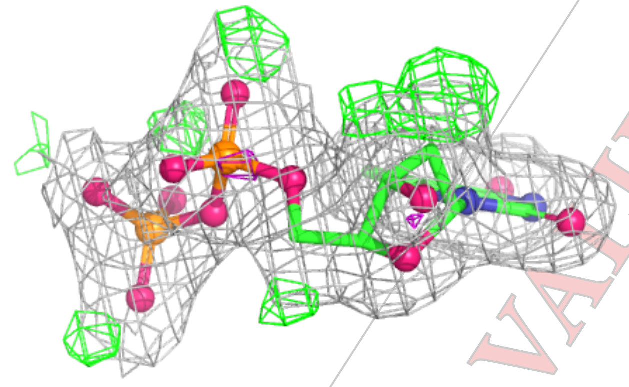

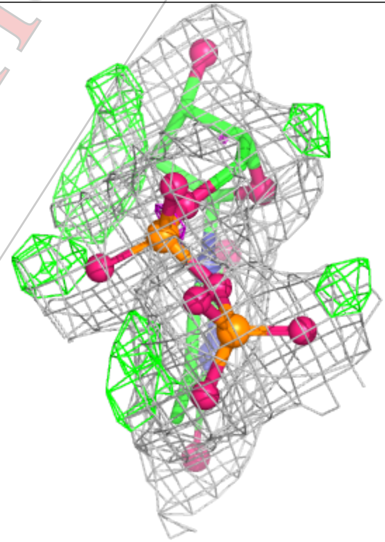

CONFIDENTIAL VALIDATION REPORT

Electron density around TYD BBB 201:

$2mF_o$-DF_c (at 0.7 rmsd) in gray
mF_o-DF_c (at 3 rmsd) in purple (negative)
and green (positive)

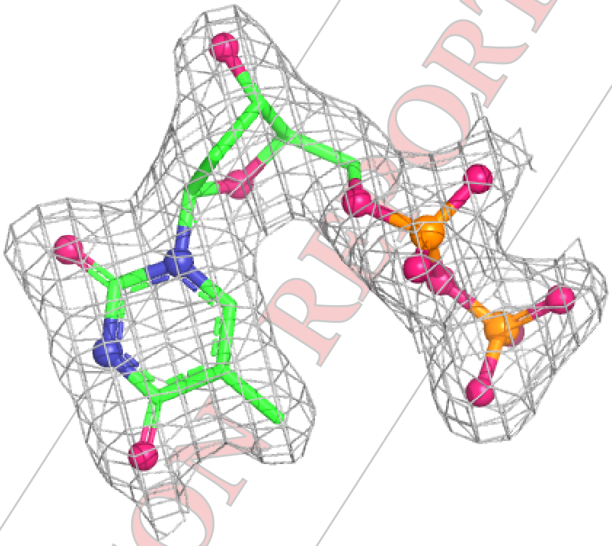

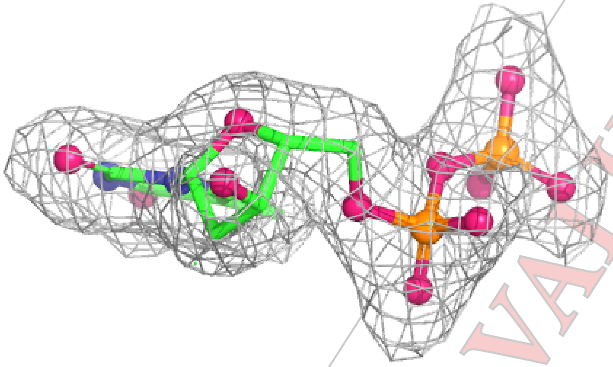

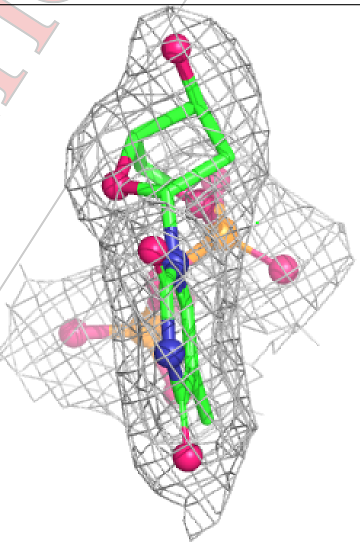

6.5 Other polymers (i)

There are no such residues in this entry.

CONFIDENTIAL VALIDATION REPORT
