# Supplementary material for: Spinning sugars in antigen biosynthesis: characterization of the Coxiella burnetii and Streptomyces griseus TDP-sugar epimerases
Source: J Biol Chem. 2022 Apr 6;298(5):101903. doi: 10.1016/j.jbc.2022.101903 (PMC9095892; doi:10.1016/j.jbc.2022.101903)
Supplement: Cross et al raw data [file mmc2.zip › ORE data drop/Summary of data.docx]

Spinning sugars in antigen biosynthesis: a direct study of the *Coxiella burnetii* and *Streptomyces griseus* TDP-sugar epimerases

Cross et al.

Overview of submitted data

Data associated with each figure and table is provided in a separate folder for ease of use. The data provided are summarised below.

Enzyme kinetic data: enzyme kinetic data are generally provided as: raw experimental data; curated experimental data for determination of the initial rate; and the interpreted initial rates for determination of kinetic parameters. Transformation of these data to provide results in different units is performed in derivative sheets. These data are provided in Graphpad format (.pzfx) with the relevant parameter fitting included, and original versions of the figures in the manuscript. The final results and images used in the paper are marked in the sheet/graph name. This file format contains all the data in a plain text XML format that can be viewed by a wide variety of programs.

Table 2:

Graphpad files for the Michaelis-Menten determination for the RmlC paralogues from *Escherichia coli* (Ec), *Streptomyces griseus* (Sg), *Coxiella burnetii* (CBU1838) and *Amycolatopsis orientalis* (Ao). These data give the *k_cat_* and *K_M_* values, from which the specific activity *k_cat_* / *K_M_* is calculated. The same data were used to generate Figure S1.

Table 3:

Graphpad file for the NMR study. The sheets provide the raw data; a normalisation of these data; and the interpreted figures for the rate derived from the normalised data. The raw images that were used to generate figures are named as in the manuscript.

Figure 2:

Provided are the alignment file used as an input to EspriPT; and the raw alignment TIFF file that was produced by EspriPT and then annotated.

Figure 3:

Provided are an Excel file containing the raw data, and a Graphpad file with the data summaries and statistical tests.

The Excel file has three tabs: RawData has the areas under the curves for each compound in each sample tested, including control and standard samples. CalCurves shows the concentration calibration using standards. Concs provides the interpreted compound concentrations and normalisation of the internal standard.

The Graphpad file contains the normalised concentrations of the compounds of interest as presented in the figure; and separate datasets for each compound to allow the statistical tests to be performed.

Figure 4:

Provided is a Graphpad file containing the raw data and the original image for the figure.

Figure 5:

Provided are Graphpad files for panels A and B. The file for panel A contains the raw NMR data, the interpreted data, and the original figure images. These data were also used to generate Figure S3.

The file for panel B contains the raw 18 hour data from NMR, the normalised data, and the original figure image.

Figure 6:

Provided are Pymol session files for panels A, B, and D; and the original Ligplot images for panels C and D. The images were prepared by hiding all but one structure as necessary. Note that to obtain the colours shown, the colour-blind friendly colour palette python file should be downloaded from <https://pymolwiki.org/index.php/Colorblindfriendly> (last accessed 10th December 2021).

Figure S1: the data are the same as those used to generate Table 2

Figure S2:

Provided is a Graphpad file containing the raw data and the original image used to generate the figure.

Figure S3: the data are the same as those used to generate Figure 5A.

Figure S4:

Provided is a Graphpad file containing the raw data and the original image used to generate the figure.

Figure S5:

Provided is a Pymol session file used to generate the image in Figure S5. Note that to obtain the colours shown, the colour-blind friendly colour palette python file should be downloaded from <https://pymolwiki.org/index.php/Colorblindfriendly> (last accessed 10th December 2021).

DNA constructs:

This folder contains files showing the full sequences for all the constructs used in this study. Files are provided in Genbank (.gb) and Snapgene (.dna) format for all constructs.

PDB deposition reports:

PDF format reports produced by the PDB for the four submissions associated with this manuscript.
