# Supplementary figures and images for: Spinning sugars in antigen biosynthesis: characterization of the Coxiella burnetii and Streptomyces griseus TDP-sugar epimerases
Source: J Biol Chem. 2022 Apr 6;298(5):101903. doi: 10.1016/j.jbc.2022.101903 (PMC9095892; doi:10.1016/j.jbc.2022.101903)

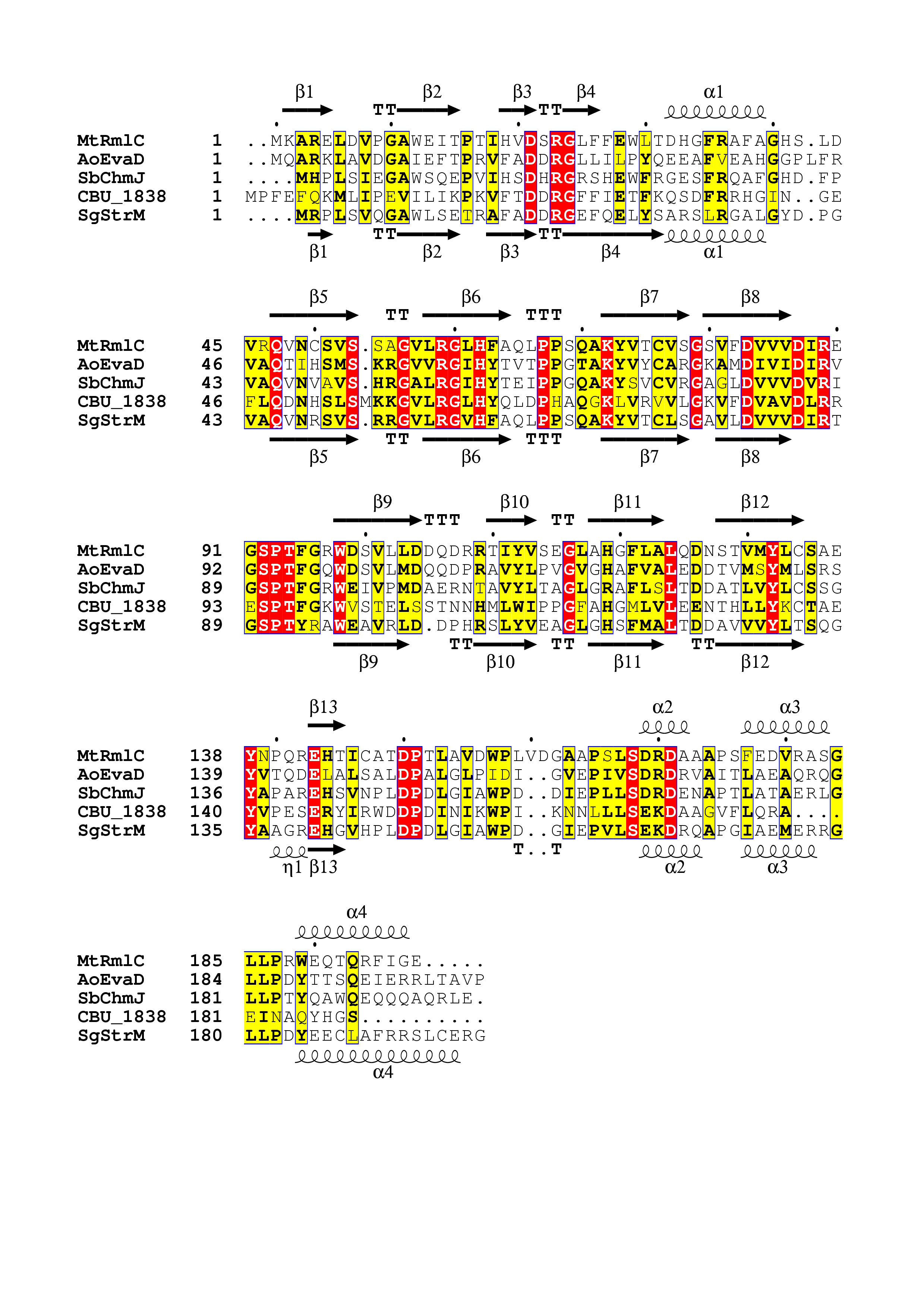

Supplement: Cross et al raw data [file mmc2.zip › ORE data drop/Figure 2/EspriPT alignment-with 2ixc and alic3.tiff]

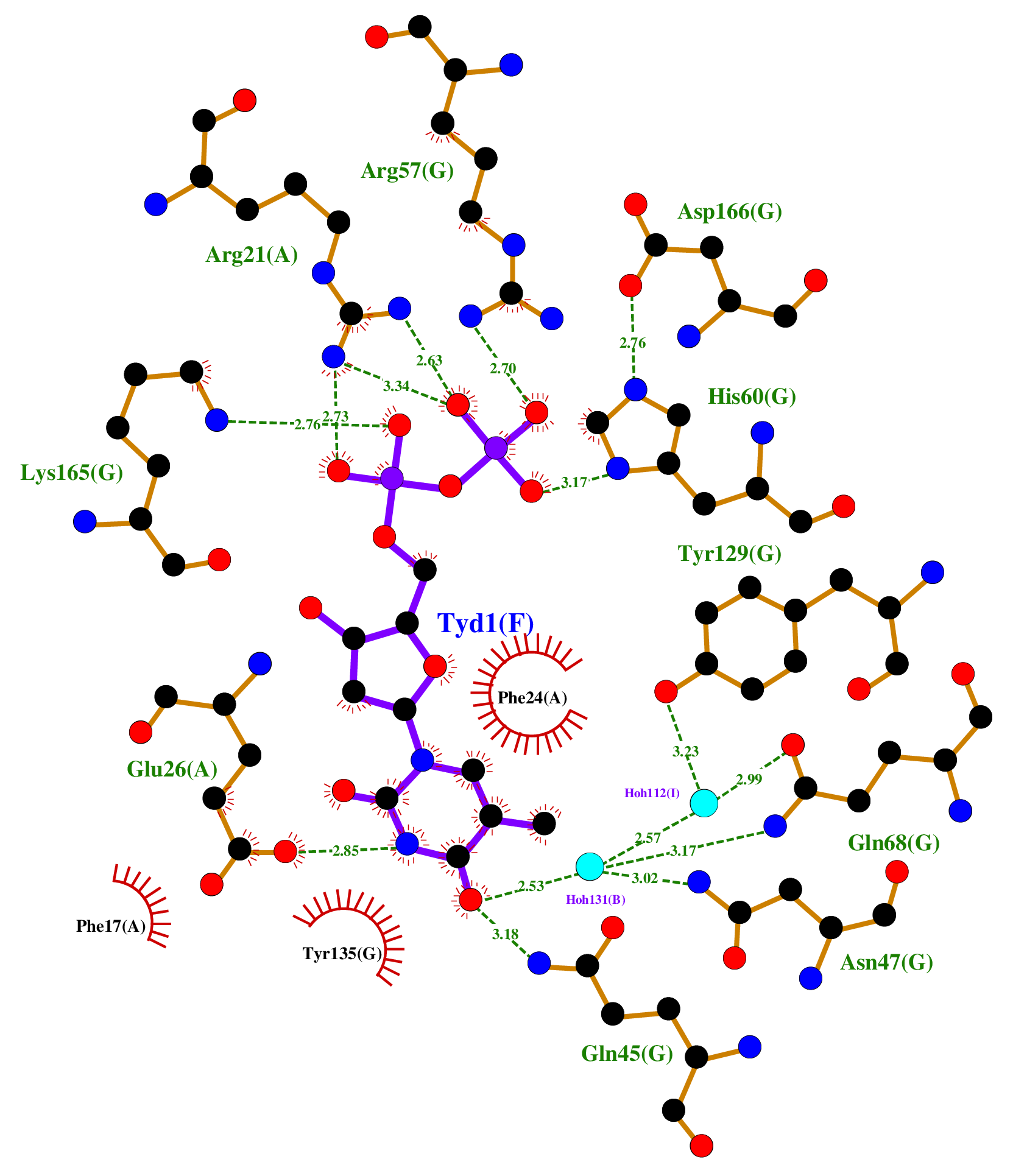

Supplement: Cross et al raw data [file mmc2.zip › ORE data drop/Figure 6/Fig6C1.png]

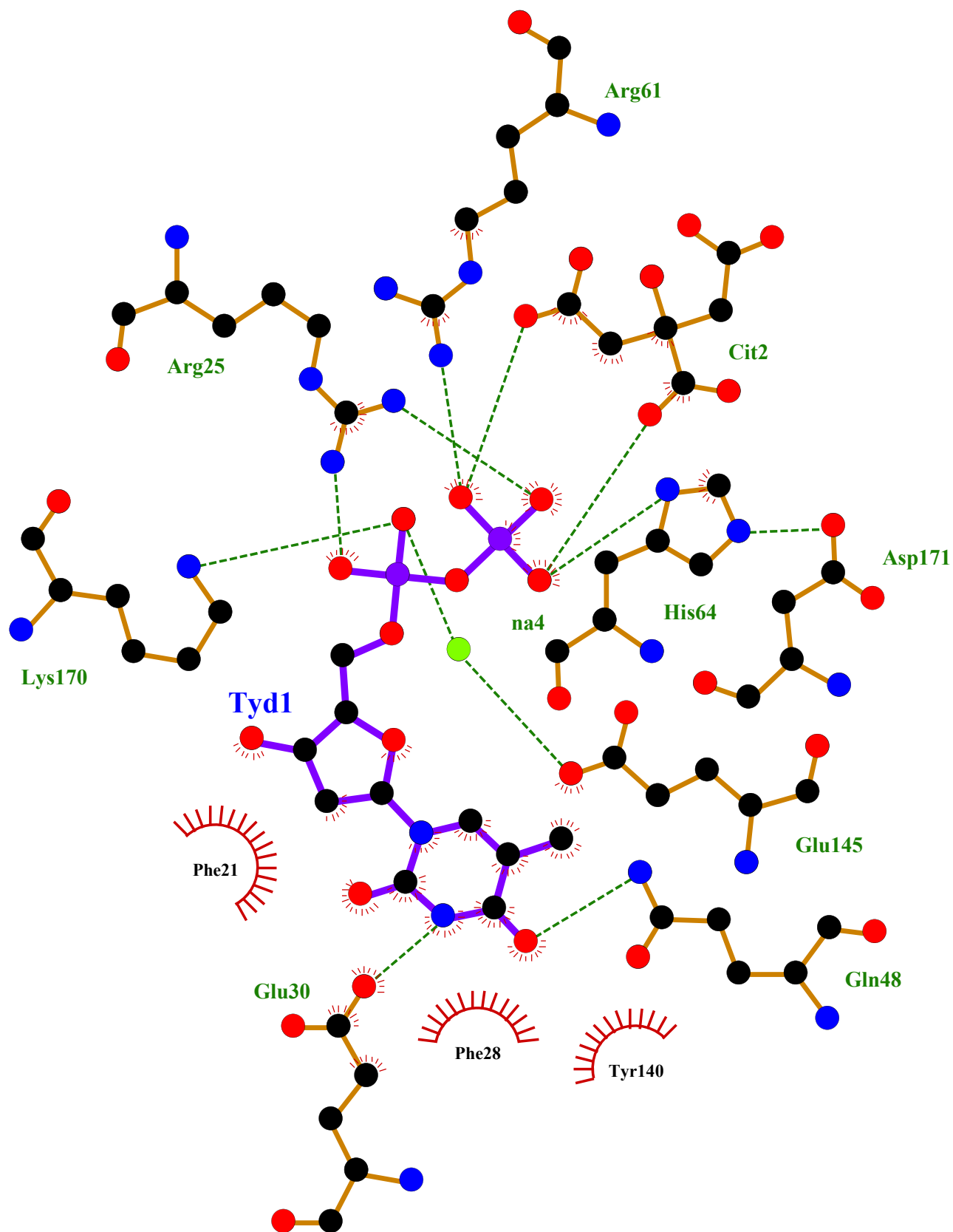

2ixi

Supplement: Cross et al raw data [file mmc2.zip › ORE data drop/Figure 6/Fig6C2.pdf]

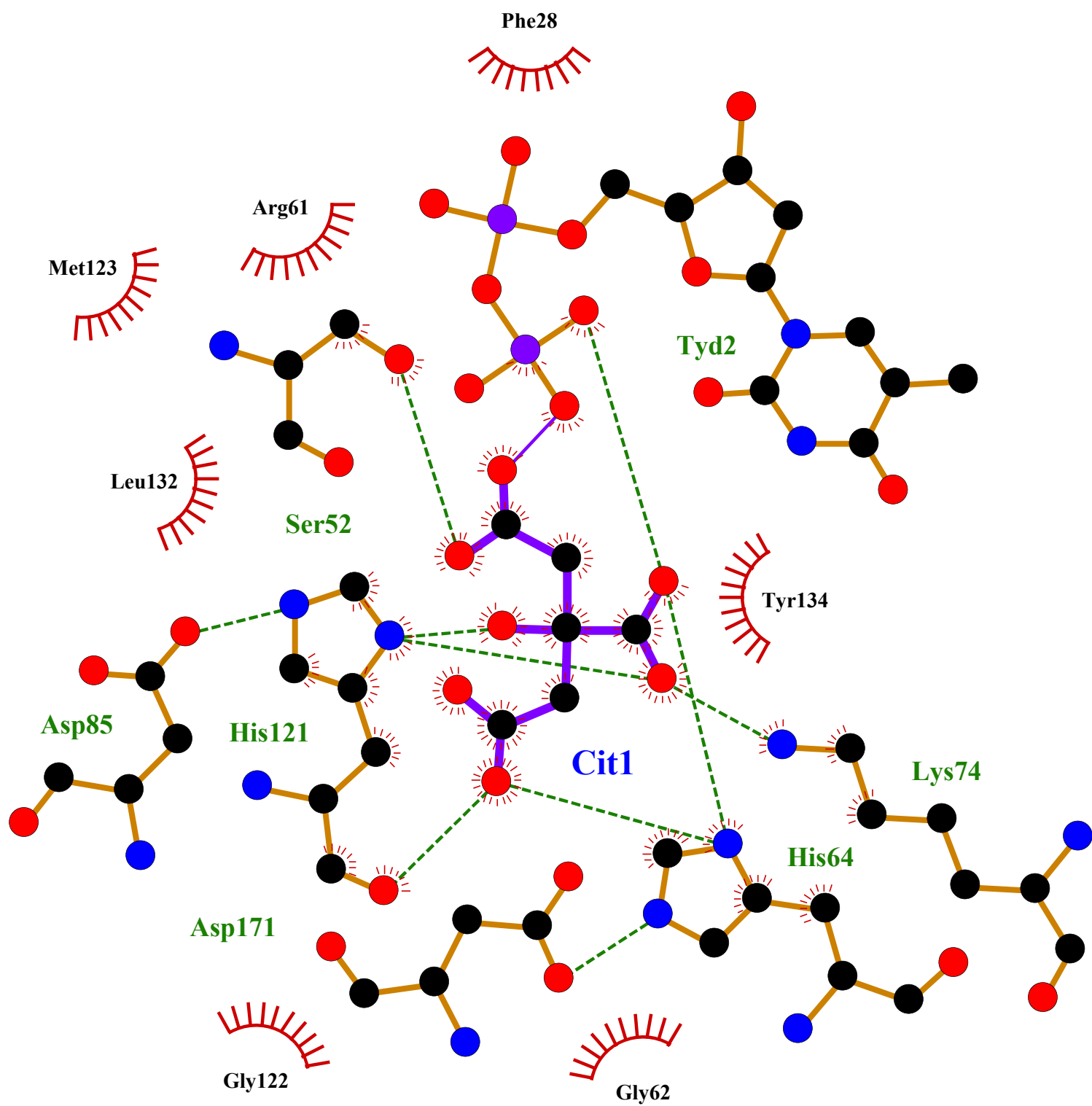

2ixi

Supplement: Cross et al raw data [file mmc2.zip › ORE data drop/Figure 6/Fig6D2.pdf]
